# High-Performance Lithium-Ion Batteries with High Stability Derived from Titanium-Oxide- and Sulfur-Loaded Carbon Spherogels

**DOI:** 10.1021/acsami.3c16851

**Published:** 2024-01-26

**Authors:** Behnoosh Bornamehr, Stefanie Arnold, Chaochao Dun, Jeffrey J. Urban, Gregor A. Zickler, Michael S. Elsaesser, Volker Presser

**Affiliations:** † INM - Leibniz Institute for New Materials, Campus D2 2, 66123 Saarbrücken, Germany; ‡ Department of Materials Science & Engineering, Saarland University, Campus D2 2, 66123 Saarbrücken, Germany; § The Molecular Foundry, Lawrence Berkeley National Laboratory Berkeley, Berkeley, California 94720, United States; ∥ Chemistry and Physics of Materials, University of Salzburg, 5020 Salzburg, Austria; ⊥ Saarene - Saarland Center for Energy Materials and Sustainability, Campus C4 2, 66123 Saarbrücken, Germany

**Keywords:** sulfur loading, hybrid carbon spherogels, carbon encapsulation, lithium-ion batteries, anode materials, electrode design

## Abstract

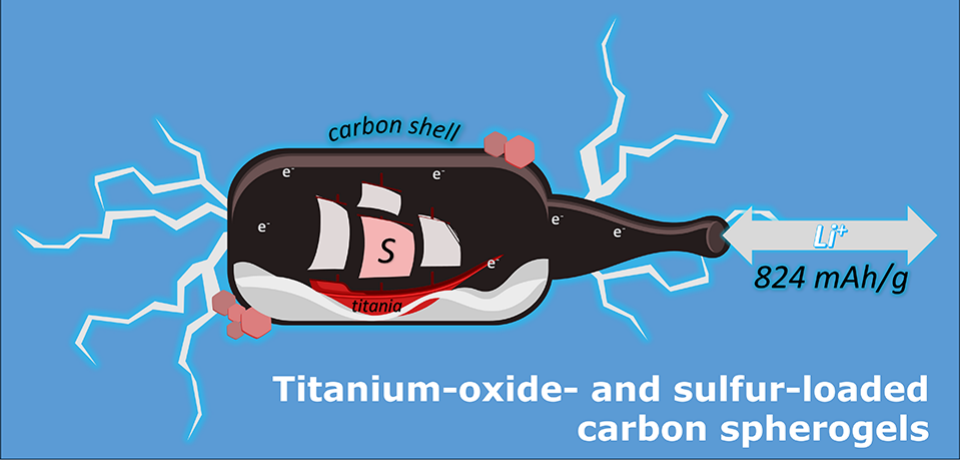

This study presents a novel approach to developing high-performance lithium-ion battery electrodes by loading titania-carbon hybrid spherogels with sulfur. The resulting hybrid materials combine high charge storage capacity, electrical conductivity, and core-shell morphology, enabling the development of next-generation battery electrodes. We obtained homogeneous carbon spheres caging crystalline titania particles and sulfur using a template-assisted sol-gel route and carefully treated the titania-loaded carbon spherogels with hydrogen sulfide. The carbon shells maintain their microporous hollow sphere morphology, allowing for efficient sulfur deposition while protecting the titania crystals. By adjusting the sulfur impregnation of the carbon sphere and varying the titania loading, we achieved excellent lithium storage properties by successfully cycling encapsulated sulfur in the sphere while benefiting from the lithiation of titania particles. Without adding a conductive component, the optimized material provided after 150 cycles at a specific current of 250 mA g^–1^ a specific capacity of 825 mAh g^–1^ with a Coulombic efficiency of 98%.

## 1 Introduction

Lithium-ion batteries (LIBs) provide effective energy storage for an array of applications, such as electric vehicles, mobile communication, and stationary energy storage units.^1,2,3^ However, the current generation of LIBs is limited by energy density, lifespan, and safety.^4^ To satisfy the growing need for high-performance batteries, the development of new materials and inventive approaches is essential. These advancements aim to enhance various aspects of battery performance, such as energy density, lifecycle durability, safety, and environmental sustainability.^5,6^ Therefore, developing new electrode materials, electrolytes, and cell designs is crucial to advancing the technology of LIBs and promoting their sustainable and efficient use in various applications. In searching for novel and sustainable materials for rechargeable battery electrodes, titania appears attractive due to its lack of toxicity and biocompatibility in contrast to cathode elements based on Ni or Co, which show severe environmental drawbacks.^7^ Early research on titania and other transition metal oxides demonstrated these materials’ benefits (and limitations) as a Li-ion battery anode.^8−10^ Unlike the widely used graphite anodes,^11^ titania has low electric conductivity, an unfavorable ionic diffusion path, and low specific capacity.^10,12^ One approach to increase the capacity of metal oxides is to convert them into their sulfide analogues. Titanium sulfide was therefore researched and reported as an attractive battery material due to its high energy density, among the early research on transition metal sulfides.^13^ As with many other sulfide compounds, a capacity loss in titanium sulfide has been a reoccurring issue and was investigated and improved to some extent by different approaches, such as hybridization with multiwalled carbon nanotubes (MWCNTs),^14^ mechanical milling,^15^ or chemical tuning to produce ternary NiTi_2_S_4_.^16^ However, a severe loss compared to the initial capacity is present to this day, and retained performance values are much below the theoretical values that can be reached by titanium sulfide even under a limited potential window, usually due to structural instability and sulfur shuttling.^14−16^

An attractive approach to stabilize the electrochemical performance is the core-shell or encapsulation strategy.^17^ The core-shell nanostructure is designed to manage the volume alteration in conversion-type materials during the lithiation/de-lithiation cycles in LIB applications; this structure enables accessibility to electroactive areas and facilitates the path for electron/ion transport.^18^ This methodology has also improved sulfur caging and mitigated sulfur shuttling in lithium-sulfur batteries.^19−22^ Incorporating a porous carbon shell also increases the electric conductivity and the ionic diffusion ability. For this, porous carbon spherogels have recently been studied as electrode materials for supercapacitors.^23^

Extremely porous, monolithic carbon materials like carbon aerogels are attracting significant attention for various applications, including drug delivery, adsorption/separation, electrochemical energy storage methods (like supercapacitors), and capacitive deionization.^24−29^ Owing to their extensive surface area, readily accessible open pores, and high electrical conductivity, carbon aerogels exclusively made up of hollow nanospheres, known as carbon spherogels, are being rigorously researched as electrode materials in various applications, including lithium-ion and sodium-ion batteries, lithium–sulfur batteries, and electrical double-layer capacitors (supercapacitors).^30−34^ Carbon spherogels can be directly employed as free-standing, binder-free supercapacitor electrodes.^23^ Leveraging the benefits of hollow carbon sphere aerogels over traditional carbon aerogels, particularly in terms of capacitance at high rates, thick-walled carbon spherogels have been developed and have shown promising results after undergoing 10,000 test cycles.^23^ Combining the carbon spherogels in hybrid materials allows a wide range of applications for this promising material class for different batteries.^35,36^ For example, Li et al. investigated CoO-loaded graphitic carbon hollow spheres as anode material in LIBs, showing a capacity of 584 mAh g^–1^ at a rate of 0.1 A g^–1^ for 50 cycles.^30^ Gao et al. showed that MoO_2_-loaded porous carbon hollow sphere composite materials show an initial charge capacity of 574 mAh g^–1^ at 0.05 A g^–1^ with a retention of 111% of the capacity after an operation of 80 cycles.^37^

This study explores sulfur-enriched titania-carbon hybrid spherogels as an anode in a half-cell configuration by using a lithium metal electrode. Our work shows a highly stable capacity using a potential range of 0.01–3.0 V vs. Li^+^/Li, even without adding extra conductive carbon to the electrodes. Our findings indicate that free sulfur within the carbon shell enhances the capacity, surpassing that of the titania-carbon hybrid alone. Furthermore, we establish that titanium dioxide within the shell plays a crucial role in determining the sulfur content, thereby influencing the electrochemical performance. Concurrently, our results suggest that incorporating sulfur into the carbon shell is more effective than forming free titanium sulfide and that the robustness of the titania-loaded carbon spheres ensures high capacity retention.

## 2 Experimental Section

### 2.1 Synthesis of the Titania-Carbon Spherogels

Titanium(IV) bis(ammonium lactate) dihydroxide solution (concentration of 50mass% in water), styrene with a purity of at least 99%, polyvinylpyrrolidone with an average molecular weight of 40,000, resorcinol at 99% purity, formaldehyde solution (37% in water, stabilized with 10% methanol), and sodium carbonate (over 99.9% purity, anhydrous) were all obtained from Sigma-Aldrich and utilized as is, without any additional purification. Acetone of technical grade, with a purity above 99%, was procured from VWR. Potassium persulfate with a purity exceeding 99.0% was sourced from Honeywell Fluka.

The synthesis of hybrid, titania-imbued spherogels was based on the methodology outlined in a previous study,^38^ utilizing titanium(IV) bis(ammonium lactate) dihydroxide as a titanium source soluble in water. In this research, we produced two variants of hybrid carbon spherogels: one with a lower concentration of titania (designated as LTiC) and another with a higher concentration (termed HTiC). This was achieved by first creating a uniform aqueous colloidal solution of polystyrene (PS) spheres. Following the method described by Du et al.,^39^ involved emulsion polymerization of styrene using potassium persulfate as the initiating agent and polyvinylpyrrolidone to regulate the size. A final concentration of 9 mass% was achieved by diluting the obtained PS sphere solution (average sphere size of 246 nm, Figure S1, Supporting Information). For the sol composition of the resorcinol-formaldehyde (RF) gels, first 0.62 g of resorcinol (R) was dissolved in 25 g of the 9 mass% PS solution, followed by the addition of 2 g or 6 g of the titanium precursor titanium(IV) bis(ammonium lactate) dihydroxide, respectively. Then, 0.925 g of formaldehyde (F) was added, followed by a stirring interval of 5 min to allow homogenization.

Following the addition of 0.012 g of sodium carbonate and a stirring period of 5 min, the pH of the mixture was adjusted to 3 by using a 2 N aqueous solution of HNO_3_, after which it was stirred continuously for an additional duration of 1 h. The gelation and subsequent aging were carried out inside cylindrical glass molds at 80 °C for 7 days. After gelation, the gels underwent a solvent exchange process, where they were submerged in acetone, with fresh acetone being replaced every 24 h over 3 days. This step was crucial for removing unreacted substances and byproducts. The wet organo-gels were then subjected to supercritical drying using carbon dioxide as the drying medium at 60 °C and 11 MPa. Subsequently, the hybrid RF aerogels were carbonized in a tubular furnace under a controlled argon atmosphere at a flow rate of 75 NL h^–1^. This carbonization was conducted at 800 °C, with a heating rate of 60 °C h^-1^, and maintained at this temperature for 2 h.

### 2.2 Sulfidation Treatment

The sulfidation treatment was carried out in an H_2_S Gero tube furnace at 650 °C for 1 h (samples denoted by -S) with a heating rate of 5 °C min^–1^ and cooling in the furnace. Before the treatment, the furnace was purged with Ar (99.999% purity) at a rate of 200 sccm (standard cubic centimeter per minute) at room temperature. The samples were then heated under a constant Ar flow of 100 sccm to 650 °C and held at this temperature under a 100 sccm flow of Ar as protective gas and 100 sccm of H_2_S as reactive gas. Prolonged sulfidation was carried out under the same treatment but with a holding time of 2 h (HTiC-2H-S).

### 2.3 Preparation of Activated Titania-Carbon Spherogels

For comparison of the performance of spherogels with and without sulfur, the spherogels were also activated under CO_2_ flow at 800 °C for 35 min directly after carbonization without further sulfur loading (denoted as LTiC-A and HTiC-A).

### 2.4 Material Characterization

Scanning electron microscopy (SEM) and elemental analysis using energy-dispersive X-ray spectroscopy (EDX) were performed with a ZEISS GEMINI 500 microscope equipped with an EDX detector from Oxford Instruments. For these analyses, an acceleration voltage of 1 kV was used for imaging purposes and 15 kV was employed for spectroscopy. These procedures were conducted on the samples before and after electrochemical testing. The samples were prepared by mounting them on an aluminum stub using double-sided copper tape. To ensure a comprehensive elemental analysis, a minimum of 20 random points on each sample were selected for point elemental analysis, with the average quantities of the detected elements being subsequently calculated. The elemental mapping focused specifically on titanium (Ti), oxygen (O), and sulfur (S).

Transmission electron microscopy (TEM) was done using a JEOL JEM F200 microscope operated at 200 keV with a cold field emission source and a TVIPS F216 camera with a resolution of 2048 × 2048 pixels. The specimen preparation involved placing the dry powder sample onto a TEM grid with a lacey carbon film deposited on a copper grid. In the case of postmortem analysis, 400 mesh copper grids from Quantifoil were used, which were covered with a holey carbon film 10 nm in thickness. To evaluate the thickness of the carbon shell, measurements were taken from 10 different spheres using ImageJ software, and an average value was calculated based on these measurements.

For energy-dispersive X-ray spectroscopy (EDX) in scanning transmission electron microscopy (STEM) mode, we used a high-performance JEOL Centurio EDX detector. This detector is characterized by its large windowless design, spanning 100 mm^2^ with a solid angle of 0.97 s, and boasts an energy resolution of less than 133 eV. The acquisition of EDX intensity maps and spectra was carried out using a typical beam current of 0.3 nA and a beam diameter of 0.23 nm. These maps were generated by integrating the counts over specific transition lines: the C K_α_ line for carbon (integration range: 0.21–0.34 keV), the O K_α_ line for oxygen (0.46–0.59 keV), the S K_α_ line for sulfur (2.20–2.41 keV), and the Ti Kα line for titanium (4.37–4.56 keV). We also used a field emission transmission electron microscope (FETEM, JEOL 2100-F) operated at 200 keW. The latter was equipped with a high-angle annular dark-field (HAADF) detector along with an Oxford high solid-angle silicon drift detector (SDD) for X-ray energy-dispersive spectrometry (EDX).

X-ray diffraction was carried out for phase analysis using a D8 Discover diffractometer (BRUKER AXS) with a copper source (Cu Kα, 40 kV, 40 mA), a VANTEC two-dimensional detector (20° 2θ angular range), a Göbel mirror, and a 1 mm point focus. The detector was moved to four positions, with a measurement time of each 1000 s, to cover an angular range of 20–80° 2θ. The dry powder was mounted on an optical glass sample holder with a 0.5 mm deep notch for sample preparation. All scans underwent background subtraction and were normalized to (0–1).

Raman spectroscopic analysis was carried out by using a Renishaw inVia Raman microscope. This microscope was equipped with a Nd:YAG laser (532 nm). The laser power at the sample’s focal point was maintained at 0.05 mW, with a numerical aperture of 0.75. For each of the samples analyzed, spectra were recorded at five different points. The exposure time for each point was set at 30 s, and the measurements were accumulated five times to enhance reliability. The analyzed powder samples were securely affixed to glass microscope slides for stability. The spectra underwent processing for cosmic ray removal and were subsequently normalized (0–1) for consistency. To ensure accuracy, the system was calibrated with a silicon standard before and after the measurements.

A Netzsch STA 449 F3 Jupiter was used for thermogravimetric analysis (TGA) with a heating rate of 10 °C min^–1^ from 20 to 1000 °C in Ar.

CHNS-O analysis was performed on the samples before and after sulfidation to measure the amounts of carbon, sulfur, and oxygen. C, H, N, and S were analyzed on a Vario Micro Cube (Elementar). For sample weighing, the standard amount of WO_3_ was added in each case. The sample was weighed in tin boats and compressed without air. The samples were then added directly to the autosampler of the CHNS analyzer. The instrument was calibrated with sulfanilamide of different weights from the instrument manufacturer (theoretical: 16.26 mass% N; 41.85 mass% C; 4.68 mass% H and 18.62 mass% S). The daily factor was determined directly before the measurement by measuring approximately 2.5 mg of sulfanilamide five times. The combustion tube temperature was 1150 °C, and the reduction tube temperature was 850 °C. The oxygen amount was measured with a rapid OXY cube (Elementar). The samples were weighed into silver boats and compressed without air. The samples were then placed directly into the autosampler of the O-analyzer. The instrument was calibrated with benzoic acid of different weights from the instrument manufacturer (theoretical: 26.2 mass% O). The daily factor was determined directly before the measurement by measuring approximately 3 mg of benzoic acid five times. The pyrolysis temperature was 1450 °C.

X-ray photoelectron spectroscopy (XPS) analyses were conducted using a K-Alpha XPS System from Thermo Scientific. This system utilized a monochromatized Al K_α_ line as its photon source, with a photon energy (*h*ν) of 1486.6 eV. A uniform spot size of 400 μm and a constant pass energy setting were employed during the acquisition process. To neutralize any charge on the samples, a combined low-energy electron-ion flood source was utilized. Additionally, to prevent any oxidation of the samples, which could affect the results, a vacuum transfer vessel was employed during the transfer and handling of the samples. This careful handling ensured the sample integrity for accurate XPS measurement.

Nitrogen sorption analyses were conducted using an Autosorb iQ system, a product of Quantachrome, now part of Anton Paar. These analyses took place at -196 °C. Prior to the measurements, degassing of the samples was done at 250 °C for 24 h to remove adsorbed species. The determination of the specific surface area (SSA) of the samples followed Brunauer-Emmett-Teller (BET), and the quenched-solid density functional theory (QSDFT) was utilized for a more comprehensive analysis assuming slit-shaped pores. In the case of the CO_2_ sorption isotherms, we used a nonlinear density functional theory (NLDFT) slit pore model.

### 2.5 Electrode Preparation

Electrochemical characterization involved the preparation of working electrodes, both with and without conductive carbon additives. For the samples lacking conductive carbon, a mixture was prepared to contain 90 mass% of the synthesized active materials (HTiC-S, LTiC-S, HTiC-2H-S) and 10 mass% polyvinylidene fluoride (PVdF, sourced from Alfa Aesar) binder, dissolved in *N*-methyl-2-pyrrolidone (NMP, 99.9% purity, from Sigma-Aldrich). This formulation, denoted as 90 for its 90:10 ratio, followed specific mixing steps outlined below. In contrast, electrodes with a composition of 80 mass% active material, 10 mass% PVdF, and 10 mass% conductive carbon (C65, from IMERYS Graphite & Carbon) were also prepared, following a similar methodology (these samples are denoted as 80 for the 80:10:10 ratio).

The preparation process began with dry grinding of the active material powder in a mortar. The dry powder was mixed at 1000 rotations per minute (rpm) for 5 min using a SpeedMixer DAC 150 SP instrument from Hauschild. NMP was added to form a viscous paste, mixed at increasing speeds: first at 1500 rpm for 5 min and then at 2500 rpm for 10 min. Afterward, the PVdF binder solution (10 mass% PVdF in NMP) was incorporated and mixed at 800 rpm for 10 min. To ensure homogeneity, the resulting suspension was stirred with a magnetic stirrer for 12 h.

The slurry batches were then doctor-bladed on a copper foil (25 μm, MTI) using a 200 μm blade and subsequently dried in a fume hood at ambient conditions. To remove residual NMP, the electrodes were subjected to a vacuum drying process at 110 °C for 12 h. The resulting electrode sheets typically exhibited thicknesses ranging from 30 μm to 40 μm and a material loading of approximately 1.4 ± 0.4 mg cm^–2^. The total mass of the whole electrode, including the current collector, was about 26.5 ± 1 mg.

### 2.6 Electrochemical Characterization

For electrochemical benchmarking, the electrodes were shaped into 12 mm disks (with an area of 1.131 cm^2^) using an EL-CELL press punch. These disks were employed as the working electrode in a two-electrode configuration within the CR2032 coin cells. Before cell assembly, we vacuum-dried the components of the cell at a temperature of 120 °C for 12 h. The assembly of the electrochemical half-cells was carried out in an argon-filled glovebox (manufactured by MBraun), maintaining an oxygen and water vapor concentration of less than 0.1 ppm. Lithium disks, each with a diameter of 11 mm, served as both the counter and reference electrode. Two Celgard 2325 pieces, cut into 18 mm diameter and presoaked in the electrolyte solution, were used as the separator in these cells. The chosen electrolyte was a 1 M solution of lithium hexafluorophosphate (LiPF_6_) dissolved in a solvent mixture of ethylene carbonate and dimethyl carbonate (EC/DMC) in a 1:1 volume ratio obtained from Sigma Aldrich. Each coin cell was filled with 150 μL of this electrolyte solution to complete the assembly. This meticulous preparation ensured a controlled environment for reliable and consistent electrochemical testing of the cells.

Electrochemical testing was conducted within a climate-controlled environment, specifically in a Binder chamber, where the temperature was consistently maintained at +25 ± 1 °C. Cyclic voltammetry (CV) measurements were performed using a VMP multichannel potentiostat/galvanostat from Bio-Logic, paired with EC-Lab software. These measurements were carried out within a potential range of 0.01–3.0 V vs. Li^+^/Li, employing various scan rates, including 0.10 mV s^–1^, 0.25 mV s^–1^, 0.50 mV s^–1^, 0.70 mV s^–1^, 1.00 mV s^–1^, 2.50 mV s^–1^, 5.00 mV s^–1^, 7.50 mV s^–1^, and 10.00 mV s^–1^. For galvanostatic charge/discharge cycling with potential limitation (GCPL) experiments, a Bio-Logic battery cycler was utilized. Rate performance measurements were also conducted to gain deeper insights into the half-cell rate capability and responses to higher currents. These measurements were carried out in the same potential window but at different specific currents: 0.05 A g^–1^, 0.10 A g^–1^, 0.20 A g^–1^, 0.50 A g^–1^, 1.00 A g^–1^, 2.00 A g^–1^, 4.00 A g^–1^, 8.00 A g^–1^, and (reverting to) 0.01 A g^–1^. All of the applied currents and calculated capacities were referenced to the active mass of the electrodes, which comprised either 90% or 80% by mass of HTiC-S, LTiC-S, or HTiC-2H-–S. To ensure the reliability and reproducibility of the results, at least three additional cells were tested for each experiment. The data presented were based on reproducible results obtained from individual cells.

In the post-mortem analysis of the electrodes, a specific procedure was followed after the electrochemical testing. Initially, the cells were maintained at a voltage of 3.0 V for 12 h for de-lithiating. The cells were carefully disassembled within a controlled environment in a glovebox. This was done to prevent exposure to ambient air or moisture, which could alter the electrode characteristics and interfere with the analysis. Following disassembly, the electrodes were thoroughly washed using 5 mL of dimethyl carbonate (DMC; purity ≥99%, Sigma-Aldrich) to remove the remaining electrolyte salt. After washing, the electrodes were dried under vacuum at room temperature. This drying step was intended to remove any remaining solvent, ensuring that the electrodes were completely dry and suitable for subsequent analytical examination.

## 3 Results and Discussion

### 3.1 LTiC and HTiC before and after Sulfidation

**Figure 1:**
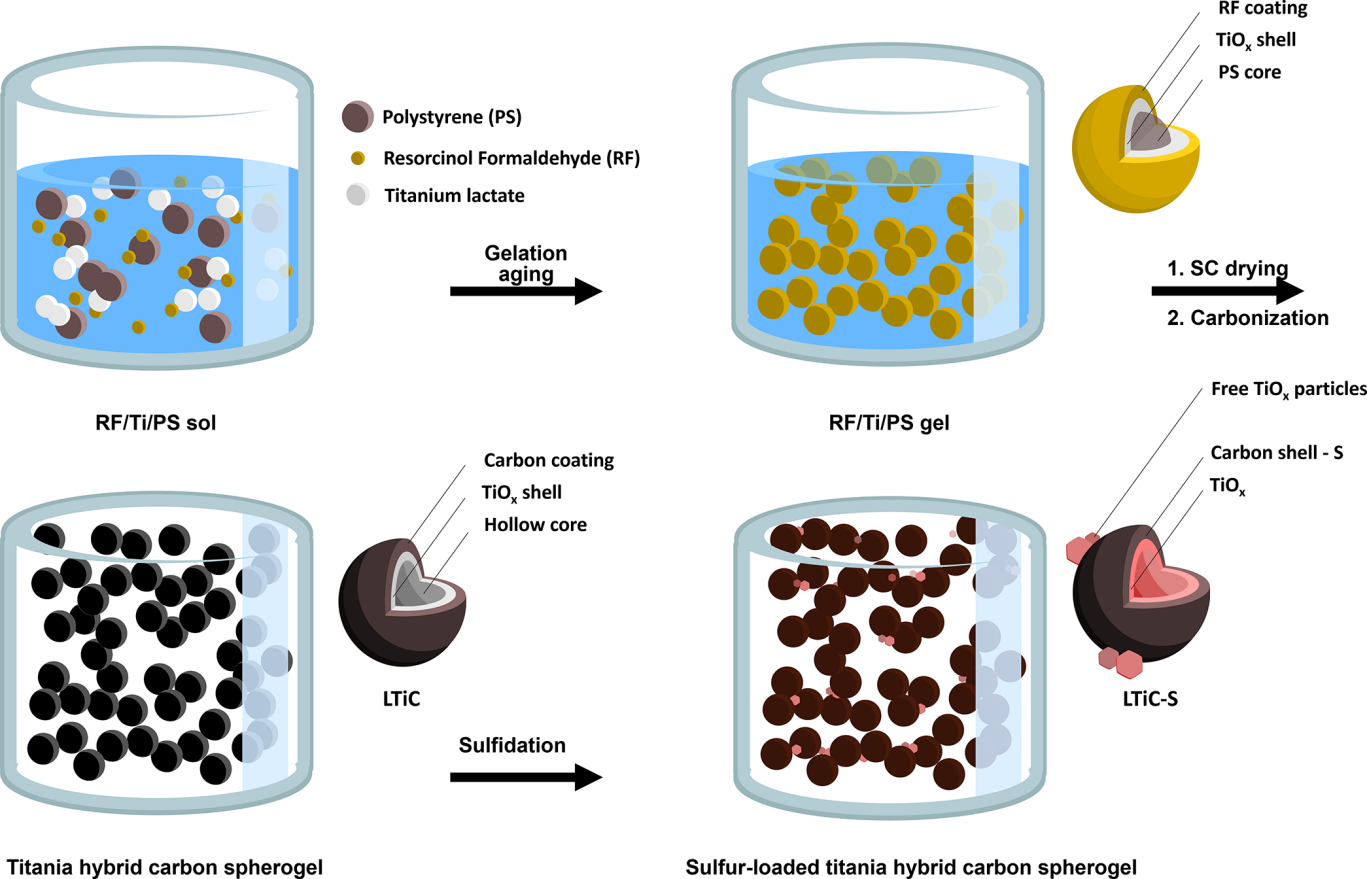
Illustrative depiction of the synthesis and processing of hybrid carbon spherogels, featuring hollow titania/carbon spheres. This process begins with polystyrene colloids serving as templates, incorporates resorcinol and formaldehyde for carbon generation, and uses titanium(IV) bis(ammonium lactate) dihydroxide as the source of titania. The term SC in this context refers to supercritical.

A schematic overview of the material synthesis and processing is depicted in Figure 1. Synthesis was carried out according to our previous report with an additional step.^38^ Using heat treatment under H_2_S gas at 650 °C for 1 h, sulfur was incorporated into the carbon shells. First, hybrid titania carbon spherogel samples were prepared via sol-gel synthesis of resorcinol and formaldehyde, templated by nanosized polystyrene spheres, and decorated with titanium lactate as Ti-source. After gelation, drying, and carbonization, one sample was loaded with a low and one with a high amount of titanium. This different mass loading of titania in LTiC and HTiC is represented by a residual titania mass of 33% and 58% after burning off the carbon shells under a synthetic air atmosphere in the temperature interval between 300 and 500 °C (Figure S1E, Supporting Information).

**Figure 2:**
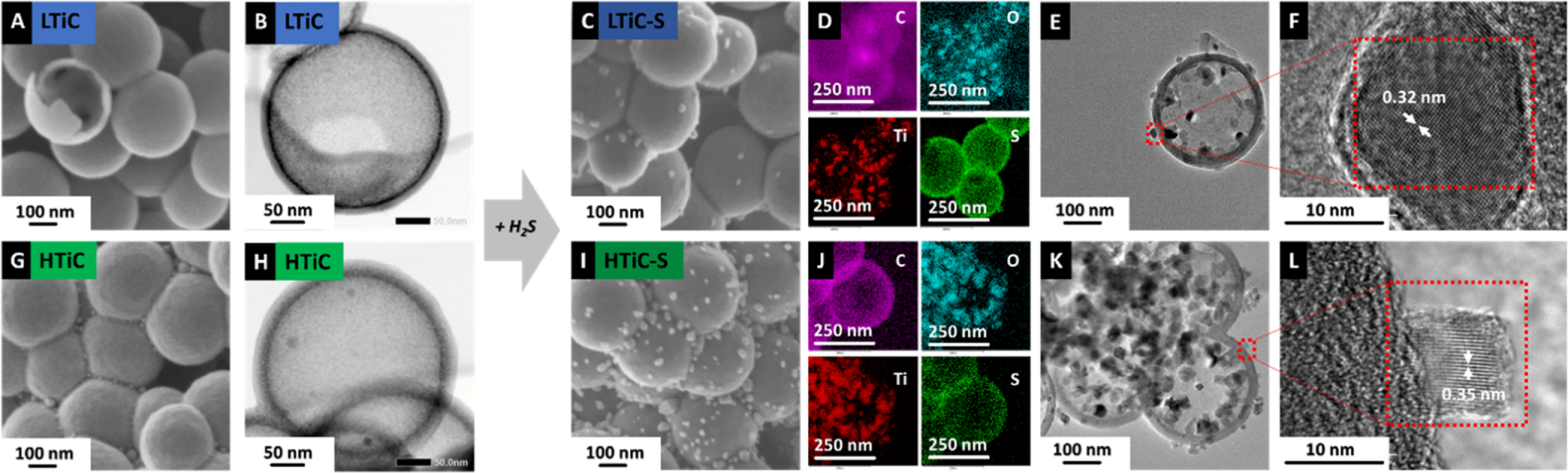
(A) Scanning electron micrograph of LTiC and (B) its corresponding STEM image. (C) Scanning electron micrograph of LTiC after sulfidation at 650 °C and (D) FETEM-EDX maps for carbon, oxygen, titanium, and sulfur elements. (E–F) Transmission electron micrographs of LTiC-S. (G) Scanning electron micrograph of HTiC and (H) its corresponding STEM image. (I) Scanning electron micrograph of HTiC after sulfidation at 650 °C and (J) elemental map (EDX) for carbon, oxygen, titanium, and sulfur elements. (K, L) Transmission electron micrographs of HTiC-S.

Scanning electron micrographs of the as-prepared titania carbon spherogels in Figure 2 show that LTiC and HTiC samples have similar homogeneous morphologies composed of spheres with a diameter of ∼300 nm. TEM depicts the homogeneous carbon shell structure of the hollow spheres with a distinct titania layer surrounded by a carbon shell with a thickness of ca. 24 nm for both LTiC and HTiC (Figure S1, Supporting Information). Higher titanium loading in HTiC is observed with extra deposition of titania between the spheres in the corners and a tighter assembly of the spheres compared to that in LTiC. After sulfidation (Figure 2C, I), the titania content and the structure of the carbon spherogel remain unchanged. In contrast, a significant increase in sulfur particle loading within and outside the spherogel is observed. Transmission electron micrographs of HTiC-S and LTiC-S show that during sulfidation, the inner titania shell grows into distinct, 10–20 nm titania crystals (Figure 2E, K). Elemental mapping of the samples by TEM-EDX shows the oxygen and sulfur distributions inside and outside the carbon sphere walls. In LTiC-S and HTiC-S, the presence of titanium and oxygen indicates titania particles, while the sulfur is mainly deposited in the carbon shell (Figure 2D, J). In the case of HTiC-S, similar to the titania distribution, there is notably more sulfur loading outside the carbon spherogel shell when compared to LTiC-S. Energy-dispersive X-ray maps of LTiC and HTiC (Figure S2, Supporting Information, and Figure S3, Supporting Information) support the statement.

**Figure 3:**
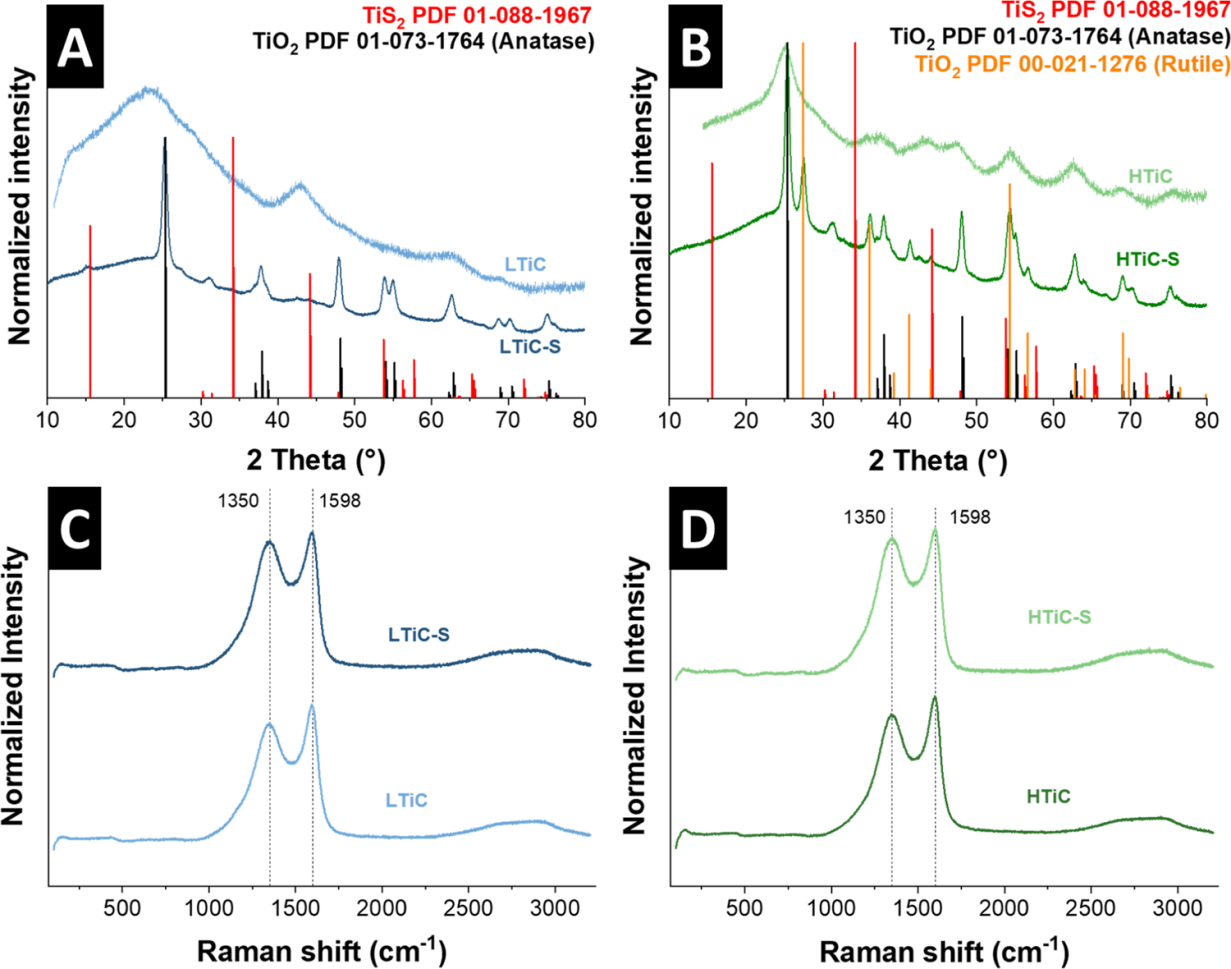
X-ray diffractogram for (A) LTiC and LTiC-S and (B) HTiC, and HTiC-S. (C) Raman spectra of LTiC and LTiC-S and (D) HTiC and HTiC-S.

X-ray diffractograms of LTiC and HTiC showed broad reflections, indicating small crystal domains before H_2_S treatment (Figure 3A, B).^40^ For the diffractogram of HTiC, a slightly higher crystallinity is observed by the distinct peaks that can be indexed with anatase. After sulfidation at 650 °C, LTiC-S and HTiC-S show high crystallinity in their structure. Strong reflections of LTiC-S can be indexed well by TiO_2_ in tetragonal lattice space group *I*41/*amd* (Figure 3A). For higher loading of titanium in HTiC-S, a lower phase homogeneity is observed. Major reflections can be indexed with titania anatase and rutile in a tetragonal lattice, space group *P*42/*mnm* (Figure 3B), indicating that the particles in the carbon shells (Figure 2C, I) are composed of titania. Although accessible to sulfidation, they crystallize in two different titania lattice structures rather than convert into titanium sulfide. This observation is validated by the identified *d*-spacings of 0.32 nm, corresponding with (110)-rutile, and 0.35 nm, corresponding with (101)-anatase (Figure 2F, L).

To monitor changes in the carbon shell upon sulfidation, Raman spectra were recorded (Figure 3C, D). LTiC and HTiC show the typical D-mode (1350 cm^–1^) and G-mode (1598 cm^–1^) of disordered graphitic carbon. After sulfidation, no changes in these bands by either a Raman shift or in the D/G intensity ratio are observed, which confirms the thermal stability of the carbon shell during sulfidation treatment and the presence of C–C bands in a mixture of sp^2^ and sp^3^ hybridization before and after sulfidation.

**Figure 4:**
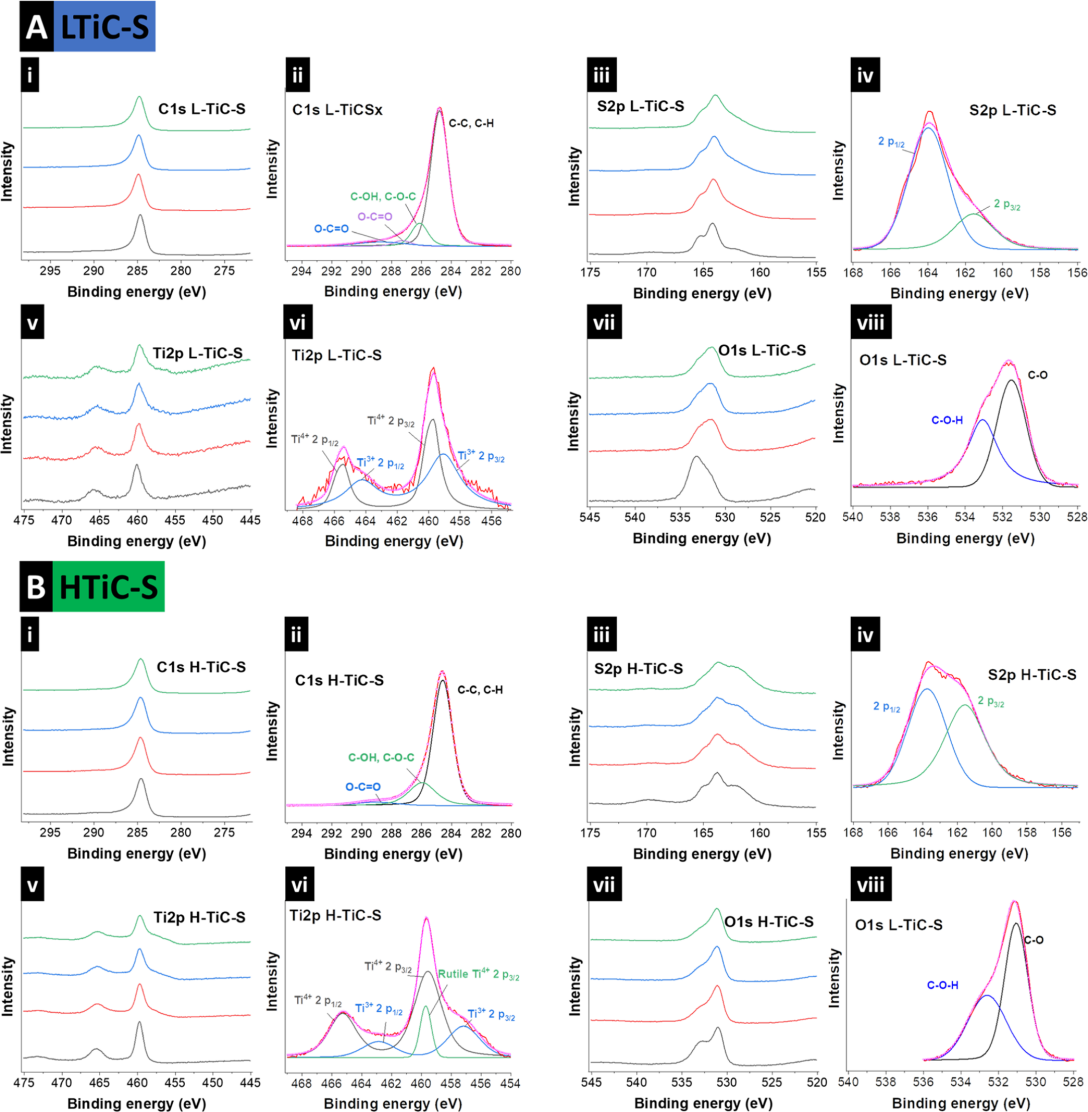
(A) X-ray photoelectron spectra using Al–Kα radiation for LTiC-S and (B) HTiC-S and separate profiles for C 1s, S 2p, O 1s, and Ti 2p, including peak fitting.

X-ray photoelectron spectra were measured for LTiC-S and HTiC-S to elucidate the nature of sulfur loading in the spheres (Figure 4), with the depth file given in Figure S4, Supporting Information. For LTiC-S, the C 1s spectrum features a distinct peak at 284.6 eV, which relates to the C–C and C–H bonding of the carbon spherogels.^41^ Surface functionalities of the carbon source can be detected in both samples with the C–OH/C–O–C and the C–C=O at 286.0 eV and 289.2 eV, respectively (Figure 4Ai+ii). The S 2p spectrum is distinguishable by its decomposition into two primary peaks at binding energies of 164 eV and 162 eV. These peaks are indicative of the 2p_1/2_ and 2p_3/2_ orbitals of elemental sulfur. The shoulder peak at 162 eV increases in intensity with progressive etching. In the etched samples, the sulfur bands exhibit similar features, with a notably broader peak around 169 eV and merged peaks observed at 162 eV, 164 eV, and 166 eV. For the Ti 2p spectra, Figure 4Avi shows two sharp bands at 466 eV and 459 eV corresponding to Ti^4+^ 2p_3/2_ and Ti^4+^ 2p_1/3_, respectively, characteristic of the main phase of TiO_2_ in the unetched patterns. O 1s scans showed an asymmetrical peak at 533 eV corresponding to C–O and C–O–H bonding in the unetched pattern that shifted to 531 eV in etched patterns (Figure 4Aviii).

XPS scans of HTiC-S showed similar behavior with a sharp carbon peak at ∼285 eV, indicating a stable carbon characteristic in both samples and the carbon shell at different depths (Figure 4Bi+ii). S 2p showed a decrease in the intensity of the 169 eV peak from unetched to the etched pattern in contrast to the LTiC-S sulfur scan, indicating less sulfur in the carbon spherogels, while an increase in the intensity of the shoulder at 162 eV with further etching was seen, indicating sulfur bonding with carbon (Figure 4Biiv+iv). Ti 2p (Figure 4Bv+vi) showed peaks and a shoulder formation at 459 eV with etching similar to LTiC-S. However, the intensity increase in the shoulder was not as substantial as that in the LTiC-S. O 1s scans presented in Figure 4Bvii–viii showed an asymmetric peak at 531 eV similar to LTiC-S with an apparent shoulder at 532.5 eV in the unetched pattern. At the same time, C–O–H reflection becomes less intense after etching. When analyzing the Ti/S ratio based on XPS, a significantly higher sulfur atomic% was obtained for LTiC-S compared to HTiC-S (LTiC-S: 92%; HTiC-S: 64%). Elemental analysis from the XPS peak deconvolution from the depth analysis in Table 1 shows that in LTiC-S, sulfur is successfully taken up by the carbon shell with a 16.6 atomic%. At the same time, in HTiC-S, this is dominated by higher oxygen amounts present in the titania.

**Table 1:** : Elemental Analysis of LTiC-S and HTiC-S from XPS Peak Deconvolution

	element (atomic%)			
	oxygen	titanium	sulfur	carbon
LTiC-S	7.6	1.43	16.6	74.4
HTiC-S	17	4.7	9.3	69

To quantify the sulfur loading, the measured amounts of C, O, and S elemental content are shown in Table 2. All of the numbers are given in mass%, and elements of carbon, sulfur, and oxygen are shown. Before sulfidation, more than half of the LTiC is composed of carbon, about 20 mass% more than HTiC due to its lower titania loading. A lower difference is seen for oxygen content, with a 7 mass% difference for LTiC and HTiC. Still, the oxygen is assumed to be bonded to titanium; therefore, a lower amount of oxygen is present in low titania loading in LTiC. After sulfidation, a lower difference in carbon content in LTiC-S and HTiC-S is observed. 10 mass% of oxygen in LTiC-S is lost, and sulfur loading is 33 mass% of the material, while in HTiC-S, nearly all oxygen is maintained. Lower than 10 mass% is sulfur loading of the spheres, confirming the XRD and EDX data on higher sulfur loading in LTiC-S, which is about three times higher than that of HTiC-S. Since it was assumed this low sulfur loading in HTiC-S was due to a higher amount of titania than LTiC-S, prolonged sulfidation was carried out for 2 h at 650 °C to encourage sulfur diffusion in the spheres. The resulting product was labeled as HTiC-2H-S. SEM and XRD data confirm the formation of titanium sulfide (Figure S5A, B, Supporting Information). Crystalline TiS_2_ reflections were observed by XRD alongside those of anatase and rutile (Figure S5B, Supporting Information). However, the formed sulfides were not homogeneous and showed high coarsening. The scanning electron micrograph shown in Figure S5A, Supporting Information, reveals that the sulfide sheets are formed outside the carbon spheres, outgrowing them with a diameter of ∼2 μm. These sheets are probably formed from free titania outside the spheres. Due to a lack of protection from the carbon shell, these sheets coarsen and grow rapidly under heat treatment. CHNS analysis showed this sample is composed of 8.8 mass% sulfur, similar to HTiC-S.

**Table 2:** : Elemental Analysis of LTiC and HTiC before and after Sulfidation by CHNS-O Analysis in Mass%

	element (mass%)		
	carbon	oxygen	sulfur
LTiC	57.61 ± 0.39	23.49 ± 0.18	
LTiC-S	39.69 ± 0.54	13.30 ± 0.74	33.21 ± 0.76
HTiC	35.19 ± 0.30	30.44 ± 0.58	
HTiC-S	30.45 ± 0.11	29.84 ± 0.36	9.30 ± 0.18

The nitrogen gas sorption isotherms (Figure S6, Supporting Information) of the untreated LTiC and the sulfidized counterpart validate that the open pore structure within the carbon spherogel remains intact after sulfur treatment. Moreover, there is no alteration in the pore volume throughout the sulfur infiltration process, even though additional mass (via sulfur) is added. This indicates that the carbon spheres maintain the shell porosity and enable ion transport for electrochemical operation.

Titania-carbon spherogels anodes without sulfur loading were tested as blank samples (LTiC-A, HTiC-A) to assess their initial performance for comparison with later sulfur-loaded samples (LTiC-S, HTiC-S, HTiC-2H–S, Figure S7, Supporting Information, and Figure S8, Supporting Information). Cyclic voltammetry (CV) was carried out in the potential range of 1.0–3.0 V vs. Li^+^/Li at various scan rates of 0.1–10 mV s^–1^. In Figure S7A, C, Supporting Information, the first cycle and every fifth cyclic voltammogram of each specific current is plotted. Both samples show a pair of pronounced and reversible reduction/oxidation peaks at 1.36 V vs. Li^+^/Li and 2.38 V vs. Li^+^/Li (values taken for 10 mV s^–1^) due to the insertion/extraction process of Li-ions from the titania lattice.^42−44^ These peaks can be attributed to the reaction mechanism in which Ti^4+^ is reduced to Ti^3+^ during discharge. In the subsequent charge cycle, the Ti-ions have oxidized again, which indicates the high reversibility of anatase TiO_2_. The complete electrochemical reaction during cycling follows the mechanism shown in eq 1.
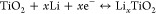
1

In the electrochemical analysis, a notable decrease in the intensities of cathodic (discharge) and anodic (charge) currents was observed at lower rates across the potential window. Additionally, there was a discernible shift in the peak maxima, with oxidation peaks moving toward higher potentials and reduction peaks shifting to lower potentials.

Galvanostatic charge and discharge experiments were conducted to characterize the electrochemical behavior and associated intercalation reactions. The reduction and oxidation peaks identified in the cyclic voltammetry experiments correlated with the galvanostatic discharge and charge profiles, as illustrated in Figure S7B, D, Supporting Information. In these experiments, both samples exhibited plateaus around 1.7 V vs. Li^+^/Li during oxidation and approximately 2.0 V vs. Li^+^/Li during reduction. Although anatase TiO_2_ possesses a theoretical specific capacity of 335 mAh g^–1^, based on its reaction mechanism with lithium, the practical capacity that can be achieved is significantly lower. This limitation is attributed to the strong Li-Li repulsion within the Li_*x*_TiO_2_ framework, which becomes more pronounced with a higher degree of lithium insertion.^45^ This phenomenon effectively limits the amount of lithium that can be intercalated into the TiO_2_ structure without causing structural instability or significant efficiency losses.

Cycling stabilities conducted at 0.1 A g^–1^ presented in Figure S7E, Supporting Information, show that less than half of the titania’s theoretical capacity is achieved with different titania loading. HTiC-A showed slightly improved capacity retention (46 mAh g^–1^, 77%) after 100 cycles, while LTiC-A outperforms slightly, achieving capacity values of 54 mAh g^–1^ and capacity retention of 70%. Most capacity fading occurred over 15 cycles, mainly influenced by solid electrolyte interphase (SEI) formation. For LTiC-A, the cycling stability is very discontinuous up to about the 160th cycle, and the Coulombic efficiency shows significant scattering. This behavior, confirmed by several cells, indicates the presence of side reactions and inhomogeneities of the synthesized material and the electrode. The rate handling ability of the titania hybrid carbon spherogel materials was investigated at different rates, that is, 0.01 A g^–1^, 0.025 A g^–1^, 0.05 A g^–1^, 0.1 A g^–1^, 0.25 A g^–1^, 0.5 A g^–1^, 1.0 A g^–1^, 2.5 A g^–1^, 5.0 A g^–1^, 10 A g^–1^, 25 A g^–1^, 0.01 A g^–1^, and finally 0.025 A g^–1^ with five cycles conducted at each specific current (Figure S9F, Supporting Information). In the last cycle of the first specific current (0.01 A g^–1^), LTiC-A and HTiC-A show capacity values of 80 mAh g^–1^ and 48 mAh g^–1^, respectively.

The hybrid materials demonstrated the anticipated electrochemical behavior, with a proportional decrease in de-lithiation capacity when subjected to higher specific currents. Upon reverting to the initial current conditions, the capacity retention for these materials ranged between 91% and 98%, with LTiC-A showing 91% retention and HTiC-A exhibiting a 98% retention rate. This decrease in lithium-ion storage capacities observed at faster charge/discharge rates can primarily be attributed to kinetic limitations. Despite this, all samples maintained stable behavior across all of the tested rates, although they exhibited lower capacities at the higher rates. In conclusion, the process of synthesizing hybrid TiO_2_/carbon materials, followed by CO_2_ etching of the carbon component, successfully resulted in electrochemical behaviors that were in line with those reported in the literature. This outcome confirms that the synthesis and modification techniques employed were effective in achieving the desired electrochemical characteristics, as referenced in the literature.^46−49^

**Figure 5:**
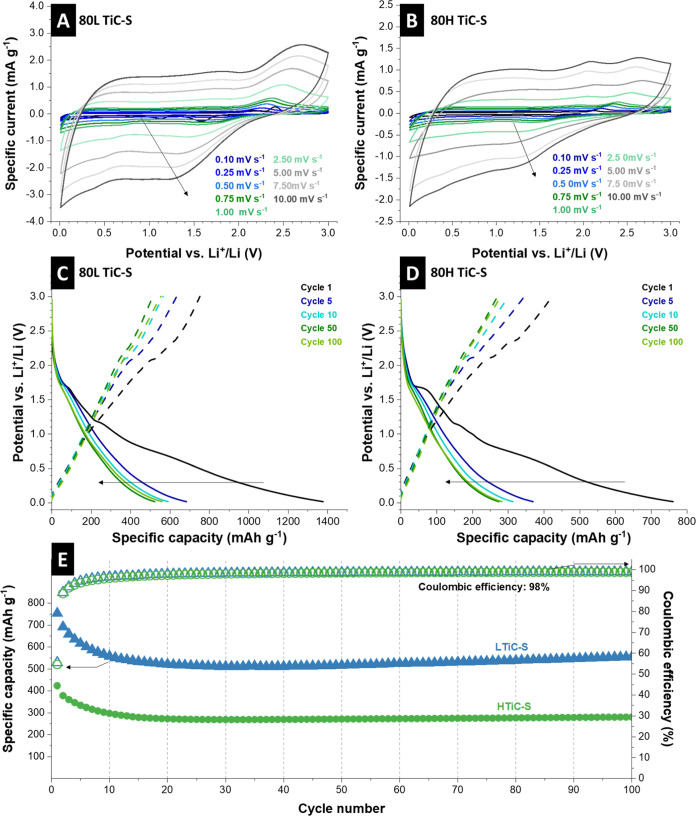
Electrochemical characterization of titania and sulfur-loaded carbon spherogels showing (A) cyclic voltammograms at different scan rates for 80LTiC-S (B) and 80HTiC-S, (C) the Galvanostatic charge and discharge profiles for 80LTiC-S and (D) 80HTiC-S and (E) the Galvanostatic charge/discharge cycling performance electrochemical stability at a specific current of 250 mA g^–1^ for 80LTiC-S and 80HTiC-S.

To overcome the limited capacity and obstacles of intercalation-type materials reported above and in the literature,^35^ sulfur was introduced, enabling electrochemical conversion reactions, thus delivering the possibility of a much higher reversible capacity. After the sulfidation process, the electrochemical performance was studied while the potential window was extended to lower values to include the conversion reaction. The obtained redox peaks in the cyclic voltammograms (Figure 5A, B) can be ascribed to the multistep reaction of intercalation and conversion while showing much higher specific capacity values than the titania-carbon spherogels.

The cyclic voltammograms of the corresponding samples, including normalization to the scan rate, are depicted in **Figure S9**, Supporting Information. In the first cycles, three reduction peaks at 1.6 V, 1.1 V, and 0.7 V vs. Li^+^/Li and corresponding oxidation peaks at 1.9 V, 2.0 V, and 2.4 V vs. Li^+^/Li are observed for both samples. The redox couple at 1.6 V and 2.0 V vs. Li^+^/Li can be attributed to the intercalation and de-intercalation of Li-ions into anatase.^12,50^ Further irreversible redox peaks in the first cycle of all samples are ascribed to the irreversible deposition of the electrolyte and SEI formation. The additional pair of sharp redox peaks indicates that during lithiation/de-lithiation, sulfur’s electrochemical reduction and oxidation occur in several stages. The peak or the shoulder at 1.6 V vs. Li^+^/Li can, besides the contribution to intercalation and de-intercalation of Li-ions, also be related to the reduction of elemental sulfur to lithium polysulfides (Li_2_S_*x*_), while further reduction to Li_2_S_2_ and Li_2_S is possible. The oxidation process includes the slow formation of Li_2_S_*x*_ at around 1.9 V vs. Li^+^/Li until elemental sulfur is produced at 2.4 V vs. Li^+^/Li.^51^ As we see, the cyclic voltammograms of the second and fifth cycles almost perfectly match. This indicates a good electrochemical reversibility of lithium-ion insertion and extraction in the samples.

Kinetic investigations were performed to characterize further possible pseudocapacitive features, including the rate-dependent analysis of the current signal. Using *i* = *av*^*b*^ to describe the relationship between the current (*i*) and scan rate (*v*), fitting parameters *a* and *b* can be evaluated. Generally, a *b*-value of 0.5 corresponds to an ideal diffusion-limited process, which typically characterizes a battery-like behavior, and a *b*-value of 1.0 indicates a surface-limited charge storage process, such as ion electrosorption.^52−54^ Figure S10, Supporting Information, provides a more detailed analysis of the *b*-values for samples with and without the addition of conductive carbon across scan rates ranging from 0.1 to 1.0 mV s^–1^. For the LTiC-S sample, a notable lithiation/de-lithiation peak exhibited a *b*-value of 0.60. A similar *b*-value of 0.61 was observed for the HTiC-S samples. These values suggest a rather battery-like insertion process of lithium ions into the anatase structure. Additionally, both samples showed more pronounced pseudocapacitive behavior at potentials of 0.5 V and 2.75 V vs. Li^+^/Li. The *b*-values for these regions were slightly higher, with LTiC-S exhibiting values of 0.99 and 0.78, and HTiC-S showing values of 0.85 and 0.70. These higher *b*-values are indicative of a significant surface-limited charge storage process. This conclusion is further supported by the nearly linear relationship between charge and cell voltage in the lithiation/de-lithiation curves, especially after the initial cycles. This linearity is a hallmark of surface-limited processes and reinforces the findings of the b-value analysis, highlighting the distinct electrochemical behaviors of the samples under study.

The reduction and oxidation peaks obtained from cyclic voltammetry of LTiC and HTiC are in alignment with the galvanostatic discharge and charge profiles shown in Figure 5C, D. In the first lithiation cycle, the main two plateaus at 1.7 V and 1.4 V vs. Li^+^/Li characterize the Li-intercalation into anatase TiO_2_ and SEI formation. The plateau at 1.4 V vs. Li^+^/Li disappears in subsequent lithiation curves. A long but undefined plateau was obtained in the de-lithiation process, combining the removal of Li from Li_*x*_TiO_2_ and the oxidation process toward elemental sulfur. Further reactions regarding sulfur interactions are likely to be superimposed by an intercalation reaction into the TiO_2_ host but still play a significant role in capacity contribution and stability. In general, it can be noted that the plateaus lose intensity during cycling.

Stability tests were performed at 0.25 A g^–1^ (Figure 5E). For LTiC-S, the initial capacity is 690 mAh g^–1^. An initial decrease in capacity to 510 mAh g^–1^ is then observed, which can be attributed to the shuttling of the free sulfur or titanium sulfide outside the spheres. Then, a stable capacity with a slight incremental slope reaches a capacity of 556 mAh g^–1^ at the 100th cycle, corresponding to an 80% capacity retention of the first cycle. The incremental slope can be associated with the converted elemental sulfur to lithium polysulfides in the lithiation/de-lithiation curve (Figure 5C).^55^ For the HTiC-S sample, a similar pattern in capacity change was observed, although with lower specific capacity values, as detailed in Figure 5E. The initial discharge cycle of this sample exhibited a capacity of 378 mAh g^–1^. Following this, there was a decrease in the capacity to 270 mAh g^–1^. After this initial drop, the performance stabilized and a discharge capacity of 280 mAh g^–1^ was maintained at the 100th cycle. This capacity represents a retention of 74% from the first cycle. Both the HTiC-S and LTiC-S materials demonstrated stable Coulombic efficiency values, averaging around 98%. The observed lower capacity in the HTiC-S sample aligns with characterization results, suggesting that its performance is predominantly due to the cycling of titania. In contrast, the LTiC-S sample, with its higher sulfur loading and formation of titanium sulfide, correlates with higher capacity values. Beyond the discharge capacity values, the high cycling stability observed in both materials underscores the crucial role of the carbon shell. This carbon shell not only aids in preserving the sulfur from shuttling effects but also acts as a cage, thereby contributing to the overall stability and efficiency of the materials. This caging effect of the carbon shell is a significant factor in the electrochemical performance of these materials, as indicated in the study referenced in the literature.^56^

**Figure 6:**
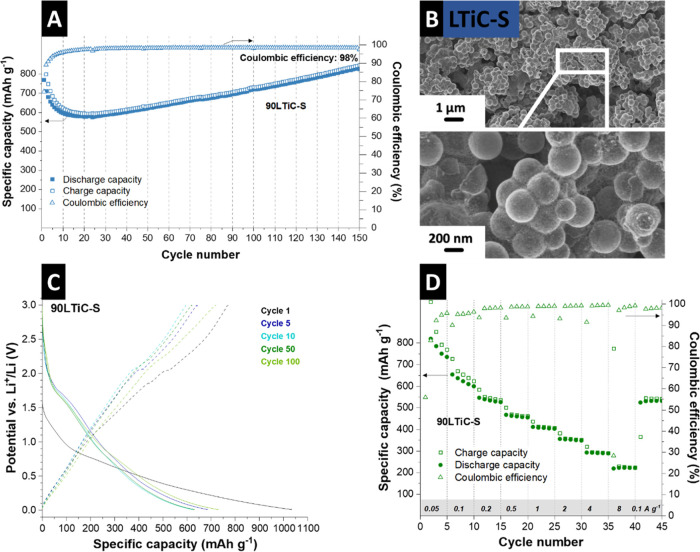
Electrochemical characterization of 90LTiC-S (90% active material plus 10% binder addition) showing **(A)** Galvanostatic charge/discharge cycling performance electrochemical stability with corresponding Coulombic efficiency, (B) scanning electron micrograph at different magnifications of post-mortem electrode after rate handling test, (C) Galvanostatic lithiation and de-lithiation profiles at an applied specific current of 0.25 A g^–1^ between 0.01 and 3.0 V vs. Li^+^/Li, and (D) rate handling ability during galvanostatic charge/discharge cycling at different rates along with the values for the Coulombic efficiency.

Prolonged sulfidation (2 h) was carried out to increase the sulfur loading (sample denoted as HTiC-2H-S). Although, in contrast to the shorter treated samples, a clear formation of titanium sulfide is detected in HTiC-2H-S, they do not perform with a high capacity due to an absence of protection of sulfur compounds by the spherogels (Figure S11, Supporting Information). The stability test shows the initial capacity is ∼680 mAh g^–1^, which falls to 300 mAh g^–1^ after 20 cycles (Figure S11D, Supporting Information). Therefore, the high capacity is not directly related to the formation of crystalline TiS_2_, but to the successful sulfur loading into the spheres and their protection by the carbon shell. Due to the conductive nature of the carbon spherogel, the LTiC-S was also tested without carbon additive to increase the active material mass loading. Results in Figure 6 (LTiC-S) and Figure S12, Supporting Information (HTiC-S) show that no extra conductive additive is needed to obtain beneficial electrochemical behavior.

Cycling stability (Figure 6A) shows that a higher active material mass loading results in stable performance. The initial de-lithiation capacity reached 768 mAh g^–1^ with a corresponding Coulombic efficiency of 89%. After an initial capacity drop in 24 cycles (575 mAh g^–1^), possibly due to structural reorganization of the carbon^57,58^ and the shuttling of the free sulfur outside the spherogel, the highly reversible capacity continuously rises throughout 150 cycles to values of 824 mAh g^–1^ with corresponding stable Coulombic efficiency of 98%. The observed increase in capacity during cycling, a phenomenon commonly reported in anode materials that contain transition metal elements, can be associated with the ongoing formation and decomposition of a polymeric gel-like layer.^59,60^ This SEI layer forms on the electrode surface and evolves dynamically during the cycling process, impacting the material’s electrochemical performance. This capacity increase is also linked to the lithiation process occurring at lower potentials, specifically around 0.01 V.^61^ Additionally, the increase in the specific surface area due to structural fragmentation during cycling may further contribute to this capacity enhancement.

A further explanation can be that sulfur doping may create carbon vacancies while reorganizing the carbon component, creating additional Li^+^ storage sites.^32,58^ It has been shown that titania acts as a host to form Li–O and Ti–S bonds.^62^ Since charge/discharge curves remain unchanged during cycling (Figure 6C), no new redox processes occur. Rather, more titania is available to host sulfur and lithium from the fragmentation of titania inside the sphere. The presence of the spherogel is essential to ensuring the reversibility of the reaction with the caged titania and sulfur. Post-mortem of the electrode after rate handling via SEM (Figure 6B) confirms that the reactions mentioned above occur in the spherogel. This is because no change in morphology has occurred, such as breaking and cracking of the spherogels or formation of secondary phases outside the cages. Post-mortem STEM-EDX confirmed that the structure stays intact and the titania particles and the sulfur-loaded carbon shell are stable (Figure S13, Supporting Information).

The charge/discharge curves (Figure 6C) with a steep slope and an indistinct plateau align with the previous profiles for the electrodes with conductive carbon (Figure 5C, D). The rate capability of electrodes without conductive carbon additives was assessed at rates from 0.05 to 8 A g^–1^, as shown in Figure 6D. These electrodes exhibited stable electrochemical behavior up to a specific current of 4 A g^–1^, with a typical pattern of decreasing de-lithiation capacities at higher currents. The capacities observed at different specific currents were as follows: 0.05 A g^–1^: The capacity was 1020 mAh g^–1^; 0.1 A g^–1^: The capacity decreased to 654 mAh g^–1^; 0.2 A g^–1^: The capacity was further reduced to 546 mAh g^–1^; 0.5 A g^–1^: The capacity was 467 mAh g^–1^; 1 A g^–1^: The capacity dropped to 411 mAh g^–1^; 2 A g^–1^: The capacity was recorded at 355 mAh g^–1^; 4 A g^–1^: The capacity further decreased to 252 mAh g^–1^: 8 A g^–1^: The lowest capacity observed was 221 mAh g^–1^. When the specific current was returned to 0.1 A g^–1^, the electrodes demonstrated high capacity retention, recovering to 89% of the initial capacity. This high retention rate indicates the robustness and stability of the electrode materials, particularly their ability to withstand various rates of electrochemical processes and then return to their original performance levels.

**Figure 7:**
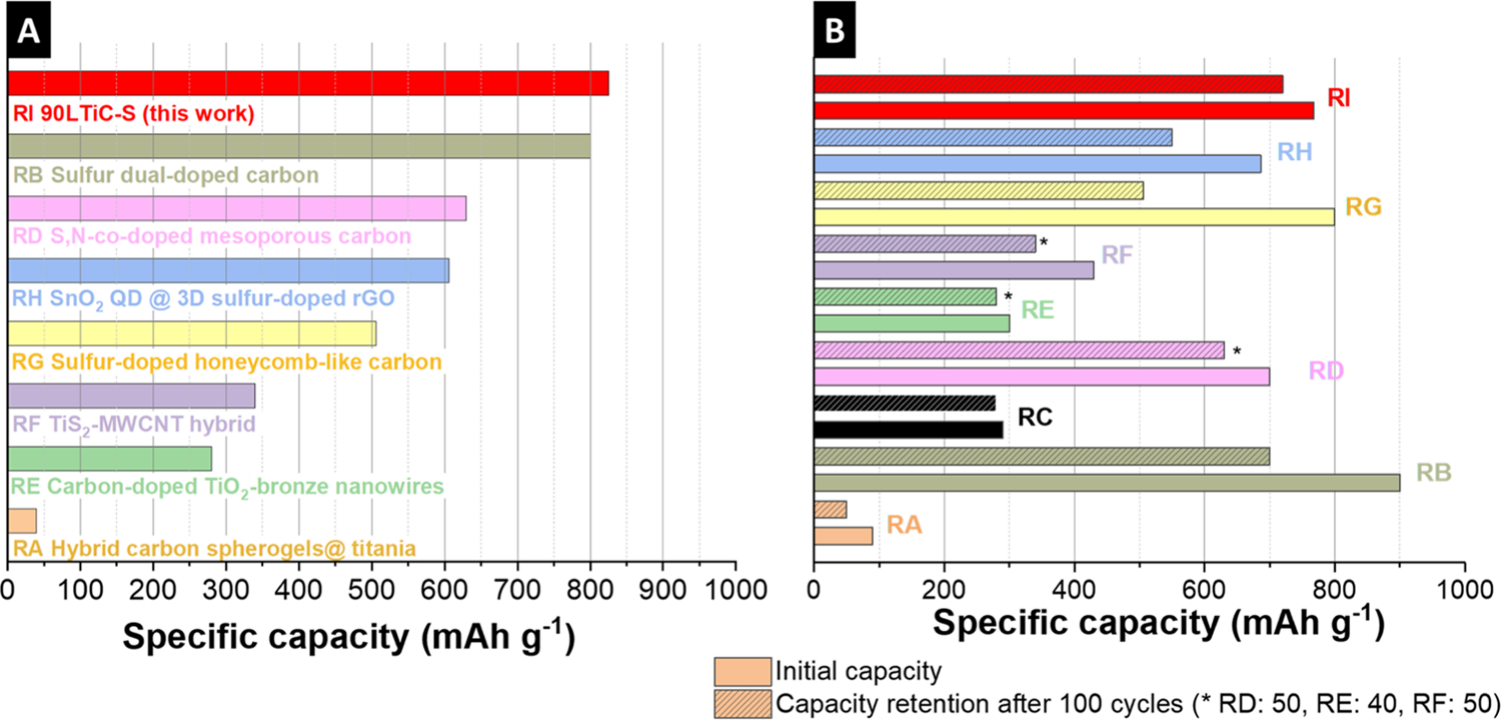
(A) Graphical illustration and overview of obtained specific capacities after cycling for different carbon, titania, titanium sulfide, and sulfur-doped materials of the state-of-the-art systems compared to the values obtained in this work. (B) Comparison of performance stability comparing initial capacity and 100th cycle capacity of different state-of-the-art systems with this work. References RA,^35^ RB,^66^ RC,^67^ RD,^68^ RE,^63^ RF,^14^ RG,^65^ RH.^64^ RI = this work.

Compared to other transition metal elements, carbon composites/hybrid materials, and sulfur-doped/based materials (Table 3, Figure 7), an optimized titania-carbon spherogel with sulfur doping was presented in this work. This feature enabled favorable electrochemical performance, which can compete with and outperform the state-of-the-art. Optimized synthesis conditions and a simple sulfidation process increase the capacity values of pure titania carbon spherogels, which deliver a capacity of 39 mAh g^–1^ at 0.1 A g^–1^ after 600 cycles.^35^ Even further developed carbon-doped TiO_2_ bronze nanowires with 280 mAh g^–1^ at 0.1C can easily be outperformed by our optimized LTiC-S sample, delivering 825 mAh g^–1^ at 0.25 A g^–1^.^63^ Also, the performance of TiS_2_-MWCNT hybrids developed by Kartick et al. with a specific capacity of 340 mAh g^–1^ at 0.1 A g^–1^ remains significantly below the values obtained in this work.^14^ The optimized material of this work is also a serious competitor with other hybrids of the conversion-type class, which, like the SnO_2_ quantum dots @ 3D sulfur-doped reduced graphene oxide, yield a capacity of 606 mAh g^–1^ at 0.5 A g^–1^.^64^ LTiC-S shows similar electrochemical behavior to sulfur dual-doped carbon films or sulfur-doped honeycomb-like carbon, which exhibit reversible capacities of 800 mAh g^–1^and 506 mAh g^–1^ (0.1 A g^–1^) over 700 and 100 cycles, respectively.^65,66^

**Table 3:** Summary of Different Lithium-Ion Battery Electrochemical Performances and Properties of Various Carbon, Titania, Titanium Sulfide, and Sulfur-Doped Materials. Data from literature sources that are not available are denoted as “n.a.”.^a^

Reference	Identifier in Figure 7	Material	Total electrode composition	Potential vs. Li^+^/Li	Electrolyte	Normalization	Capacity at rate	Cycle
Salihovic et al.^35^	RA	hybrid carbon spherogels: carbon encapsulation of nanotitania	CS-TiO_2_/C: PVdF 9:1	1.0–3.0	1 M LiPF_6_ in EC/DMC (1:1 by volume)	total hybrid	39 mAh g^–1^ at 0.1 A g^–1^	600
Ruan et al.^66^	RB	sulfur dual-doped carbon films		0.01–3.0	1 M LiPF_6_ in EC/DMC (1:1 by volume)	n.a.	800 mAh g^–1^ at 0.1 A g^–1^	700
Pappas et al.^67^	RC	heteroatom-doped carbon nanospheres	nanospheres: carbon black: PVdF 8:2:1	0.005–3.0 0.005–2	1 M LiPF_6_ in EC/EMC (3:7 by volume) + 1 mass% VC	n.a.	280 mAh g^–1^ at C/5	100
Zhuang et al.^68^	RD	S,N-co-doped mesoporous carbon	carbon: SuperP: PVdF 7:1.5:1.5	0.0–3.0	1 M LiPF_6_ in EC/DMC (1:1 by volume)	n.a.	630 mAh g^–1^ at 0.1 A g^–1^	50
Goriparti et al.^63^	RE	carbon-doped TiO_2_-bronze nanowires	nanowires: SuperP: PVdF 7:2:1	1.5–2.5	1 M LiPF_6_ in EC/DMC (1:1 by volume)	n.a.	280 mAh g^–1^ at 0.1C	40
Kartick et al.^14^	RF	TiS_2_-MWCNT hybrid	hybrid: acetylene black: PVdF 7.5:1.5:1	0.01–3.0	1 M LiPF_6_ in EC/DMC (1:2 by volume)	total hybrid	340 mAh g^–1^ at 0.1 A g^–1^	50
Wan et al.^65^	RG	sulfur-doped honeycomb-like carbon	AM: carbon black: sodium alginate 8:1:1	0.01–3.0	1.25 M LiPF_6_ in EC/DMC (1:1 by volume)	n.a.	506 mAh g^–1^ at 0.1 A g^–1^	100
Wu et al.^64^	RH	SnO_2_ quantum dots @ 3D sulfur-doped reduced graphene oxides	SnO_2_ QDs@S-rGO: SuperP: CMC 9.35:0.5:0.15	0.05–3.0	1 M LiPF_6_ in EC/DMC/DEC (1:1:1 by volume)	n.a.	606 mAh g^–1^ at 0.5 A g^–1^	500
**This work**	**RI**	**90LTiC-S**	**LTiC-S: PVdF 9:1**	**0.01–3.0**	**1 M LiPF**_**6**_**inEC/DMC(1:1 **by v**olume)**	**total mass ofcarbon+titania+sulfur**	**825 mAh g^-1^at** 0.25 A g^-1^	**150**

a EC (ethylene carbonate), EMC (ethyl methyl carbonate), DMC (dimethyl carbonates), DEC (diethyl carbonate), PVdF (poly(vinylidene fluoride)), LiPF6 (lithium hexafluorophosphate), MWCNT (multi-walled carbon nanotubes), QD (Quantum dots), rGO (reduced graphene oxide), CMC (carboxymethyl cellulose).

After optimizing the free-standing titania-loaded carbon spherogels and a simple sulfidation step, the materials obtained can be directly processed into electrodes without adding a conductive additive, resulting in a homogeneous material with better cycling performance as a lithium-ion battery anode. The improved cycling performance of titania-loaded carbon spherogels post-sulfur loading compared to initial samples results from elemental sulfur’s induction of an independent conversion-based electrochemical reaction, addressing limitations seen in intercalation-type materials, alongside potential enhancements in titania’s electrochemical accessibility due to its conversion to a more crystalline form during heat treatment. By fine-tuning further synthesis parameters and sulfidation steps, we found this hybrid material to be a promising compound for electrochemical energy storage.

## 4 Conclusions

In summary, we successfully synthesized titania-carbon hybrid spherogels with varying levels of titania loading, initially utilized without sulfidation for lithium-ion storage. The subsequent sulfidation process was aimed at enhancing the capacity either through direct sulfur loading or by forming titanium sulfide. A lower titanium loading resulted in a less crystalline titania shell, which in turn facilitated greater sulfur impregnation within the carbon spheres. This enhanced sulfur content significantly improved the lithium-ion storage properties of the material. A notable achievement of this research was the stabilization of capacity even when the potential window was extended to 0.01–3.0 V vs. Li^+^/Li. This high performance was not attributed to the highest degree of titania loading, extensive sulfide formation, or a prolonged sulfidation time. Instead, it was the effective encapsulation of sulfur within the intact, microporous walls of the carbon spheres, while still allowing for free interaction with lithium ions, that led to the observed results. Specifically, the LTiC-S electrodes achieved a high specific capacity of 825 mAh g^–1^ at a current of 0.25 A g^–1^ after 150 cycles, maintaining Coulombic efficiency values around 98% within the 0.01–3.0 V vs. Li^+^/Li potential range. These findings suggest several future research directions, such as increasing sulfur loading by reducing the crystallinity of titania or adjusting the porosity or wall thickness of the spheres. The highly adaptable nature of these hybrid carbon spherogels presents a wide array of possibilities for energy storage applications. One exciting potential avenue is their use in high-performance lithium-sulfur (Li–S) batteries, especially after exploring ways to homogeneously introduce higher amounts of sulfur into the system.

## Data Availability

The data supporting this study’s findings are available from the corresponding author upon reasonable request.

## Supporting Information Available

TEM images and TGA curves of LTiC and HTiC; elemental mapping and EDX spectra; nitrogen gas sorption isotherms of LTiC-S and HTiC-S; characterization and electrochemical performance of HTiC-2H–S; additional electrochemical analysis for LTiC-S and HTiC-S; kinetic analysis for electrode materials; electrochemical performance data for titania-carbon spherogels without sulfur treatment; additional XPS depth files and postmortem STEM-EDX (PDF)
