# Supplementary material for: High-Performance Lithium-Ion Batteries with High Stability Derived from Titanium-Oxide- and Sulfur-Loaded Carbon Spherogels
Source: ACS Appl Mater Interfaces. 2024 Jan 26;16(5):5881–95. doi: 10.1021/acsami.3c16851 (PMC10859890; doi:10.1021/acsami.3c16851)
Supplement: Supplementary file 1 — am3c16851_si_001.pdf [file am3c16851_si_001.pdf]

Supporting Information

High-Performance Lithium-Ion Batteries with High Stability Derived From Titanium-Oxide- and Sulfur-Loaded Carbon Spherogels

Behnoosh Bornamehr[1, 2, †], Stefanie Arnold[1, 2, †], Chaochao Dun[3], Jeffrey J. Urban[3], Gregor A. Zickler[4], Michael S. Elsaesser[4,*], and Volker Presser[1,2,5,*]

[1] *INM – Leibniz Institute for New Materials, Campus D2 2, 66123, Saarbrücken, Germany*

[2] *Department of Materials Science & Engineering, Saarland University, Campus D2 2, 66123, Saarbrücken, Germany*

[3] *The Molecular Foundry, Lawrence Berkeley National Laboratory Berkeley, Berkeley, CA, 94720, United States of America*

[4] *Chemistry and Physics of Materials, University of Salzburg, 5020 Salzburg, Austria*

[5] *Saarene – Saarland Center for Energy Materials and Sustainability, Campus C4 2, 66123 Saarbrücken, Germany*

* Corresponding authors:
michael.elsaesser@plus.ac.at (ME); volker.presser@leibniz-inm.de (VP)

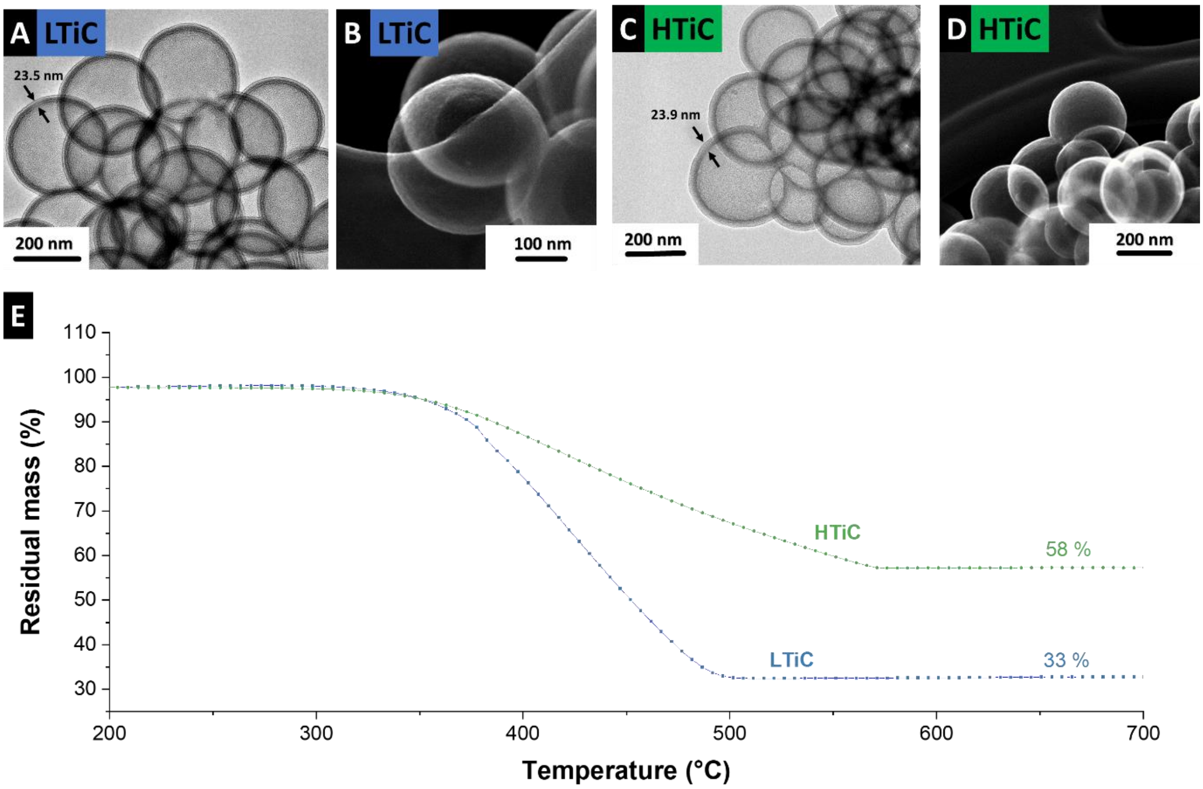

Figure S1: Transmission electron micrographs of **A-B** LTiC and **C-D** HTiC. **E** Thermogravimetric analysis (TGA) under Ar atmosphere of LTiC and HTiC.

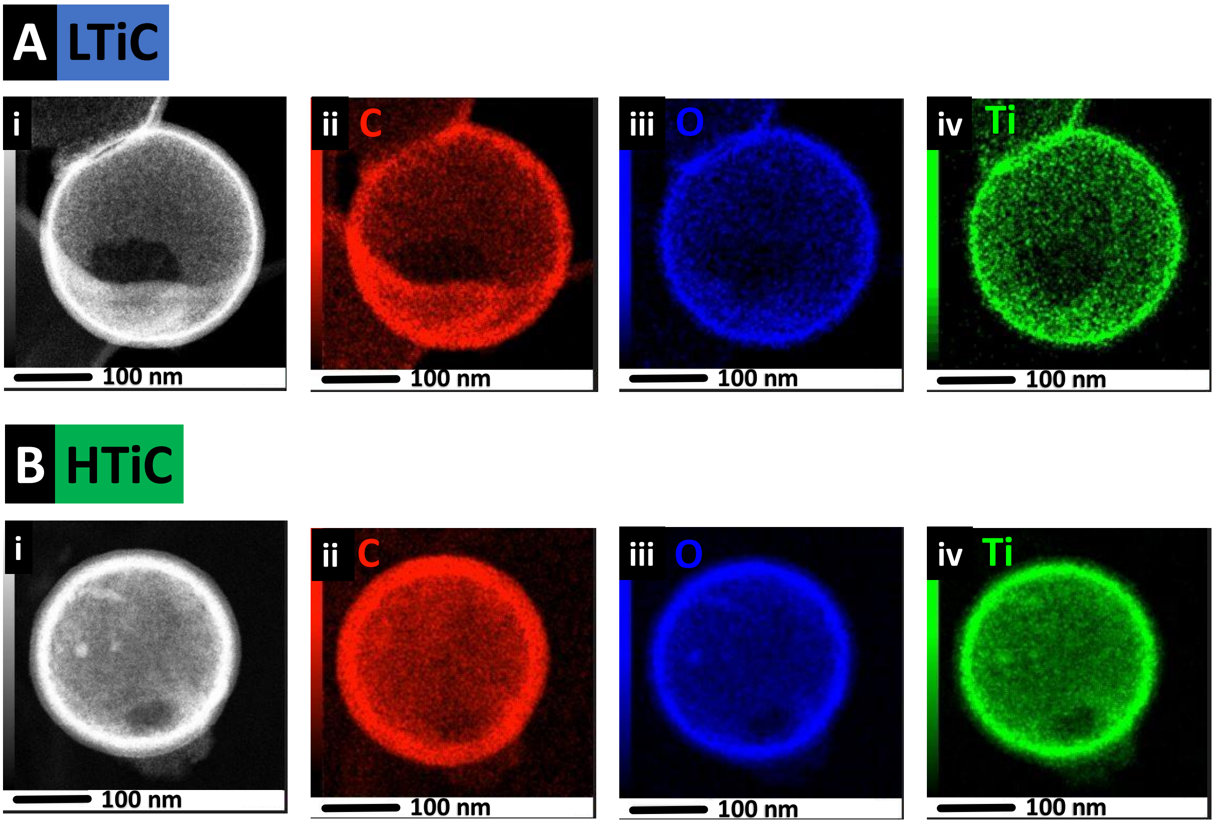

Figure S2: Energy-dispersive X-ray maps of **A** LTiC and **B** HTiC.

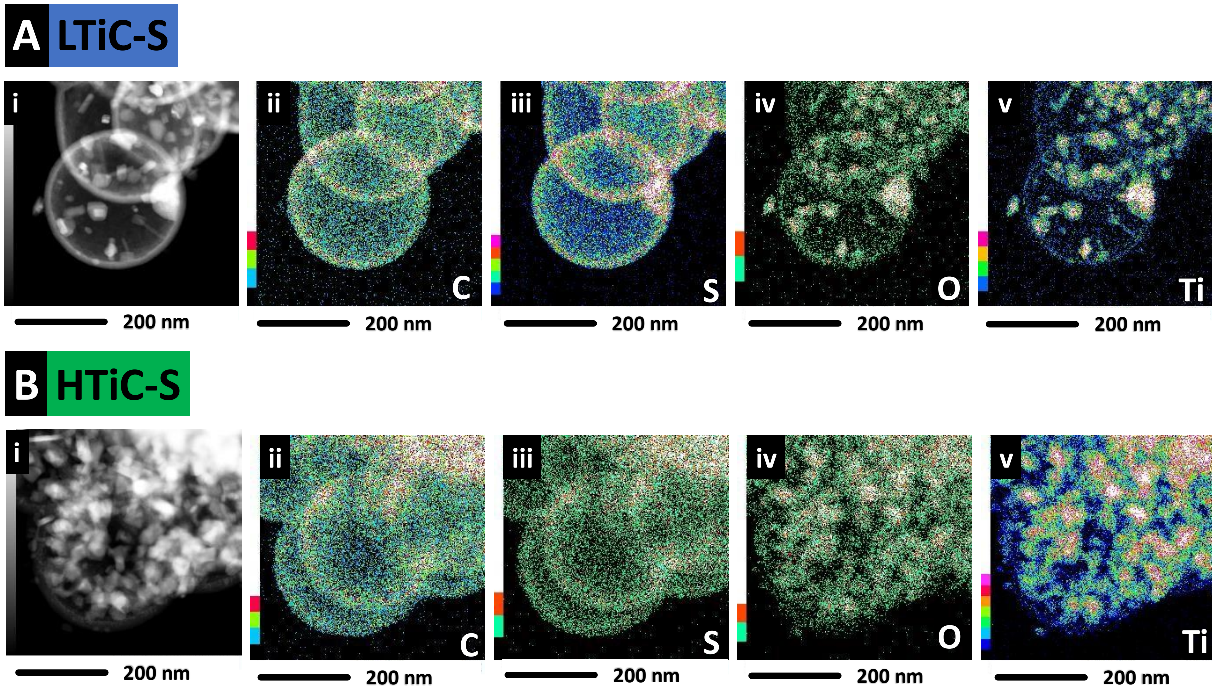

Figure S3: Energy-dispersive X-ray maps of **A** LTiC-S and **B** HTiC-S. The intensity of the maps is color-coded: colors relate to intensity, not to specific elements; colors closer to red indicate higher intensity.

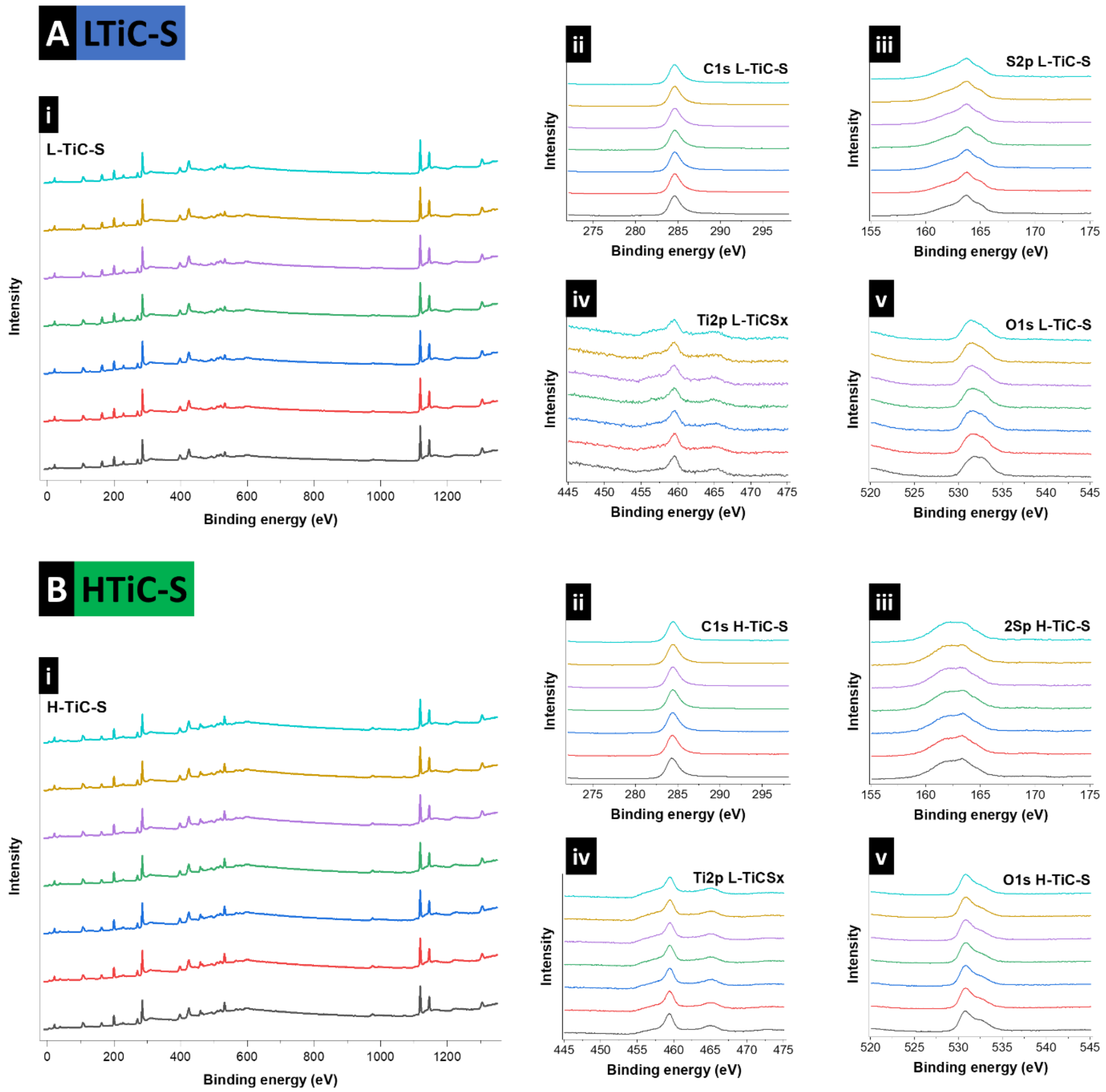

Figure S4: X-ray photoelectron spectra of C1s, S2p, Ti2p, and O1s for **A** LTiC-S and **B** HTiC-S.

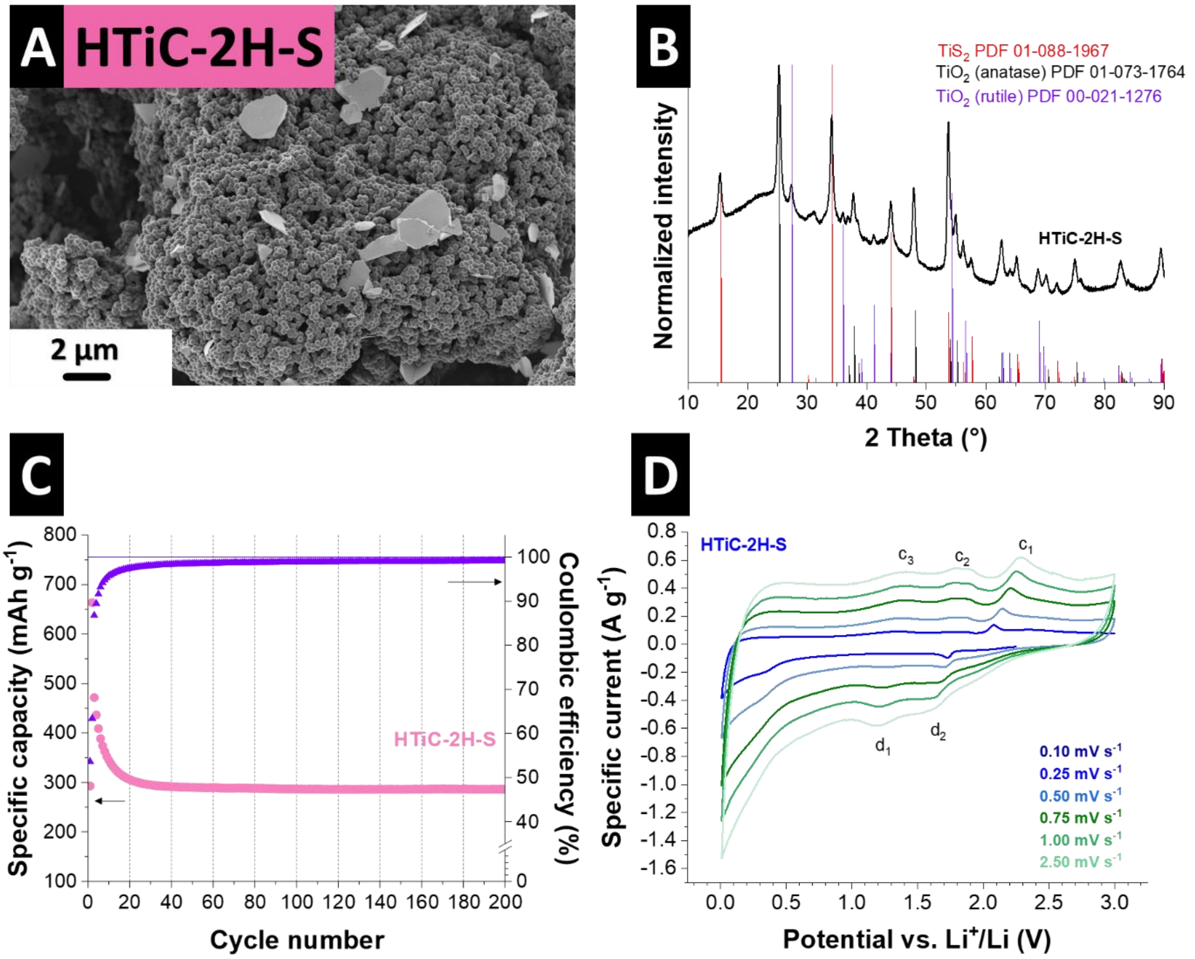

Figure S5: Characterization of HTiC-2H-S. **A** Scanning electron micrograph, **B** X-ray diffractogram, **C** electrochemical cycling stability at a specific current of 0.25 A g^{-1} including the Coulombic efficiency, **D** cyclic voltammograms at different scan rates.

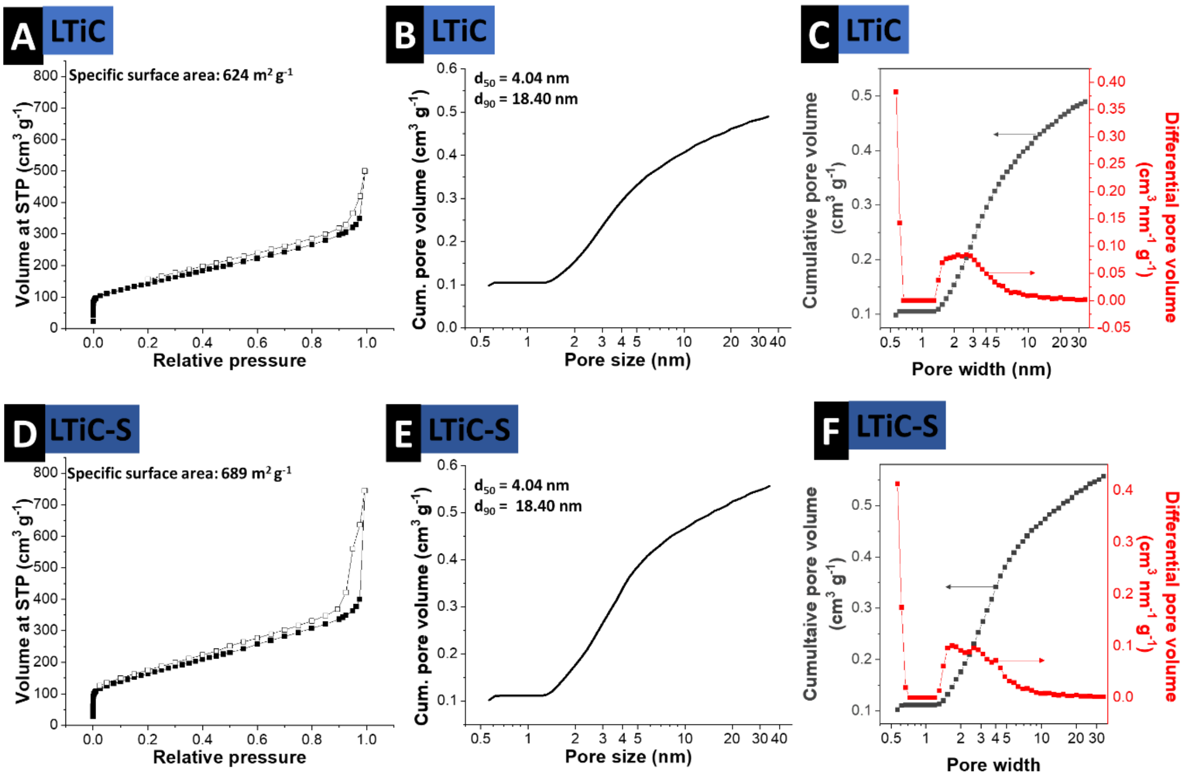

Figure S6: Nitrogen gas sorption isotherms recorded at -196°C of (A) LTiC and (D) LTiC-S. Pore size distribution pattern for (B-C) LTiC and (E-F) LTiC-S using QSDFT and assuming slit-shaped pores. STP: standard temperature and pressure.

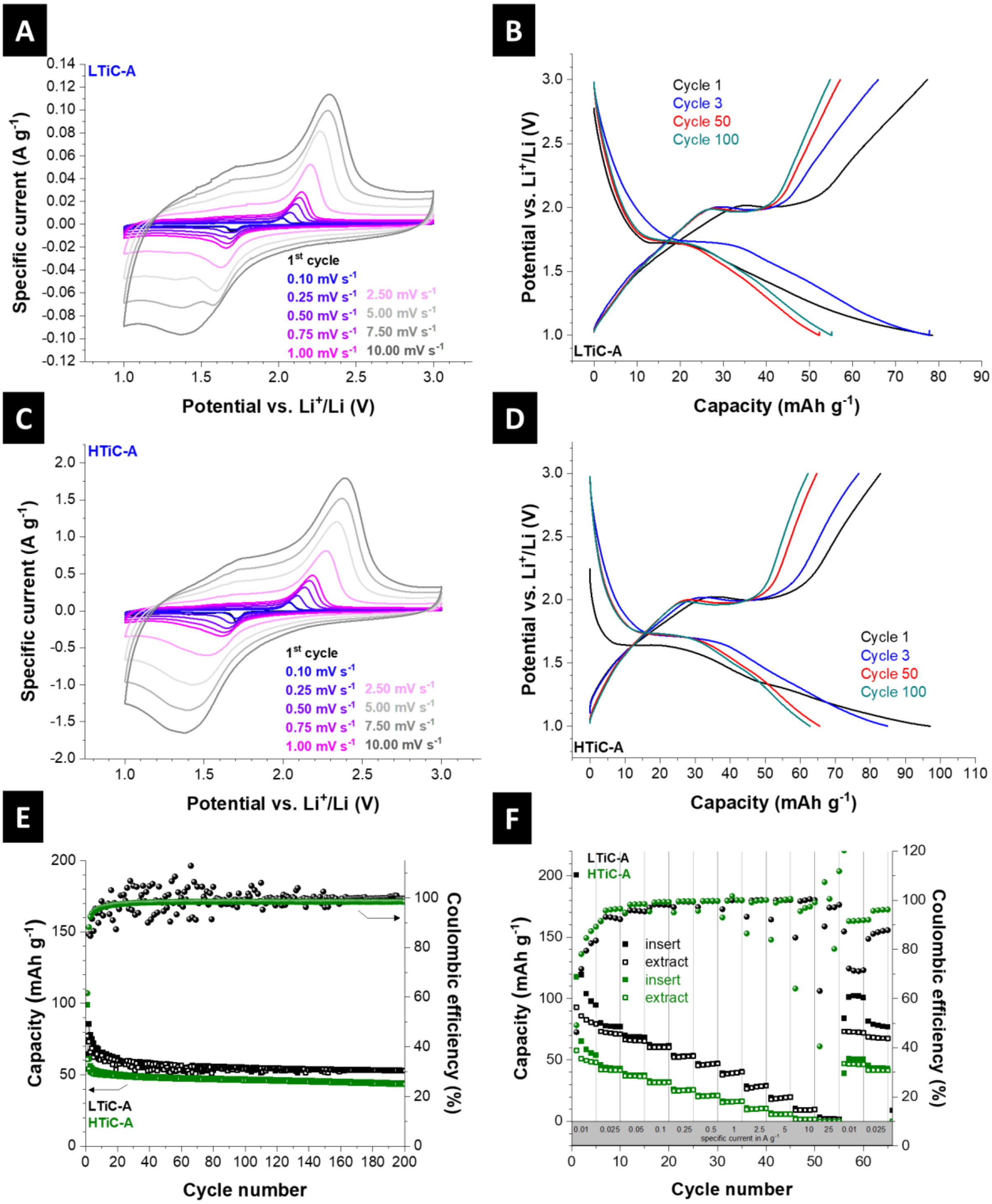

Figure S7: Electrochemical performance of different titania hybrid carbon spherogels. Cyclic voltammograms at different scanning rates and potential range between 1.0 V and 3.0 V vs. Li^+/Li and their respective charge-discharge curves at an applied specific current of 0.1 A g^{-1} for **A-B** LTiC-A, and **C-D** HTiC-A. **E** Galvanostatic charge/discharge cycling performance electrochemical stability with corresponding Coulombic efficiency at a specific current of 0.1 A g^{-1} for LTiC-A, and HTiC-A. **F** Rate performance using galvanostatic charge/discharge cycling with corresponding Coulombic efficiency at different values for the specific current for LTiC-A, and HTiC-A

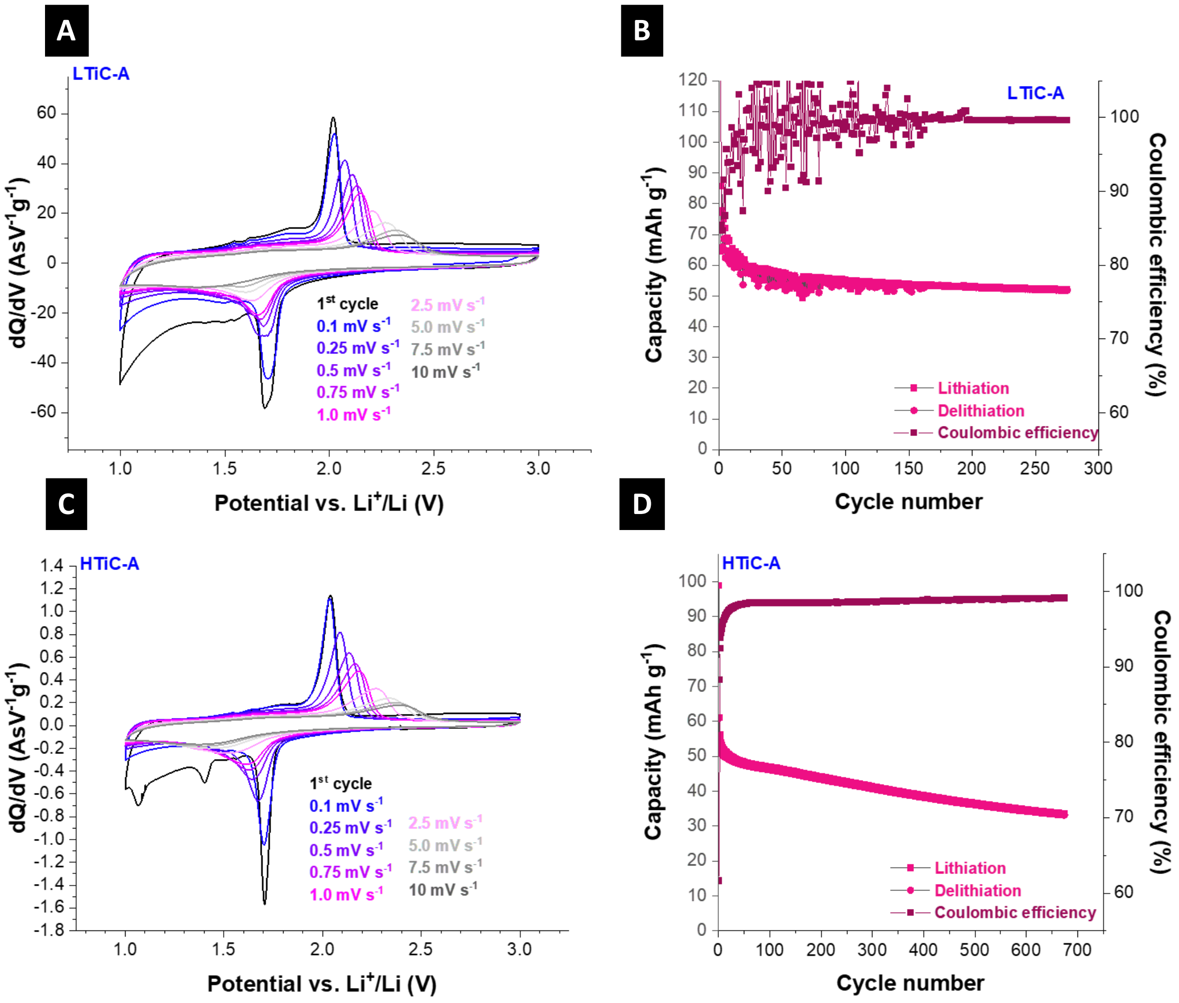

Figure S8: Electrochemical performance of different titania hybrid carbon spherogels. Cyclic voltammograms (dQ/dV) at different scanning rates ranging from 0.1 mV s^{-1} to 10 mV s^{-1} from 1.0 V up to 3.0 V vs. Li^+/Li and their respective electrochemical stability with corresponding Coulombic efficiencies at a specific current of 0.1 A g^{-1} for **A-B** LTiC-A, and **C-D** HTiC-A.

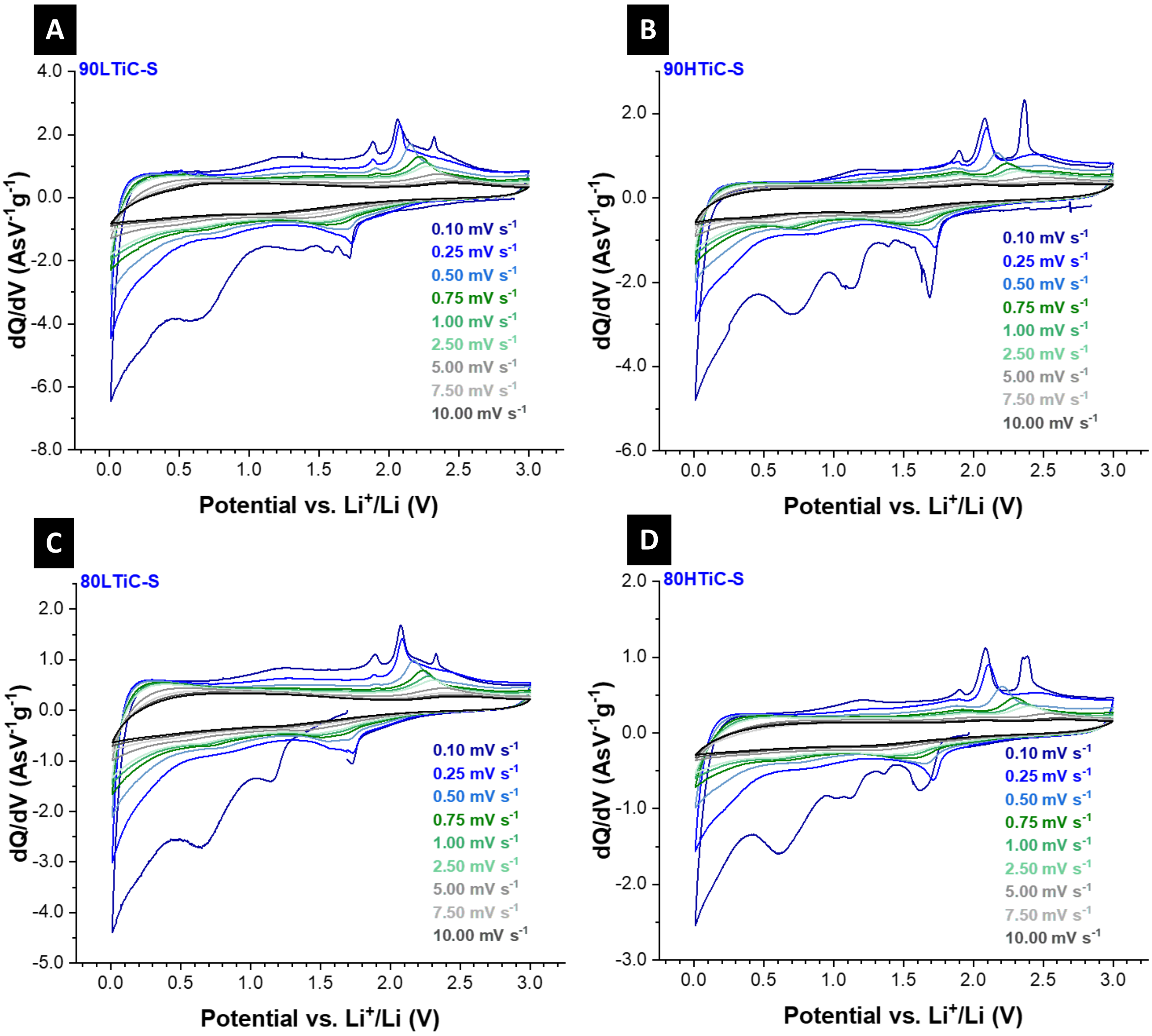

Figure S9: Cyclic voltammograms at different scanning rates and a potential range between 0.01 V and 3.00 V vs. Li^+/Li with normalization to the scan with and without the addition of conductive additive for 90LTiC-S (**A**), 90HTiC-S (**B**), 80LTiC-S (**C**), and 80HTiC-S (**D**).

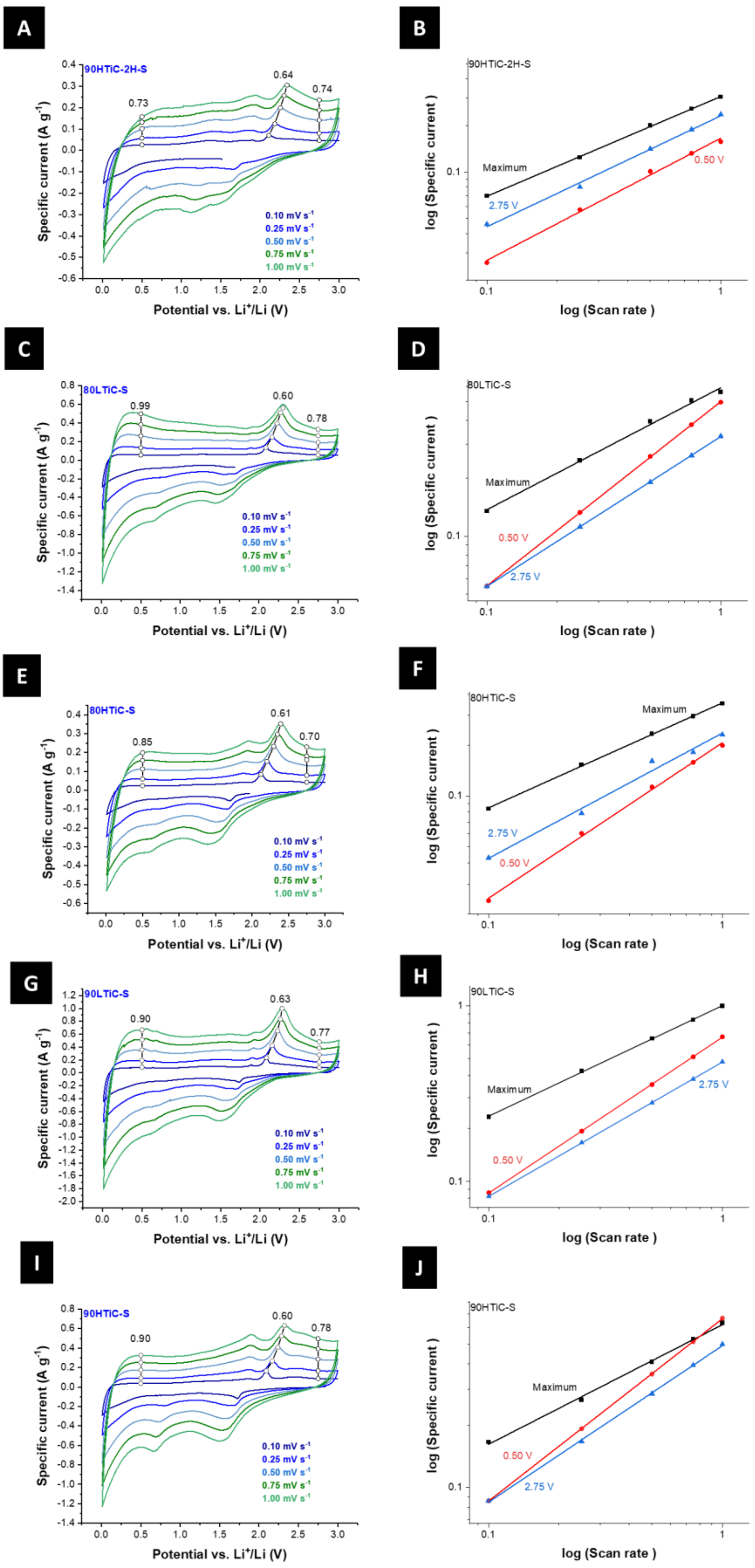

Figure S10: Cyclic voltammograms at different rates and kinetic fitting to calculate b-values for 90HTiC-2H-S (**A-B**), 80LTiC-S (**C-D**), 80HTiC-S (**E-F**), 90LTiC-S (**G-H**), and 90HTiC-S (**I-J**).

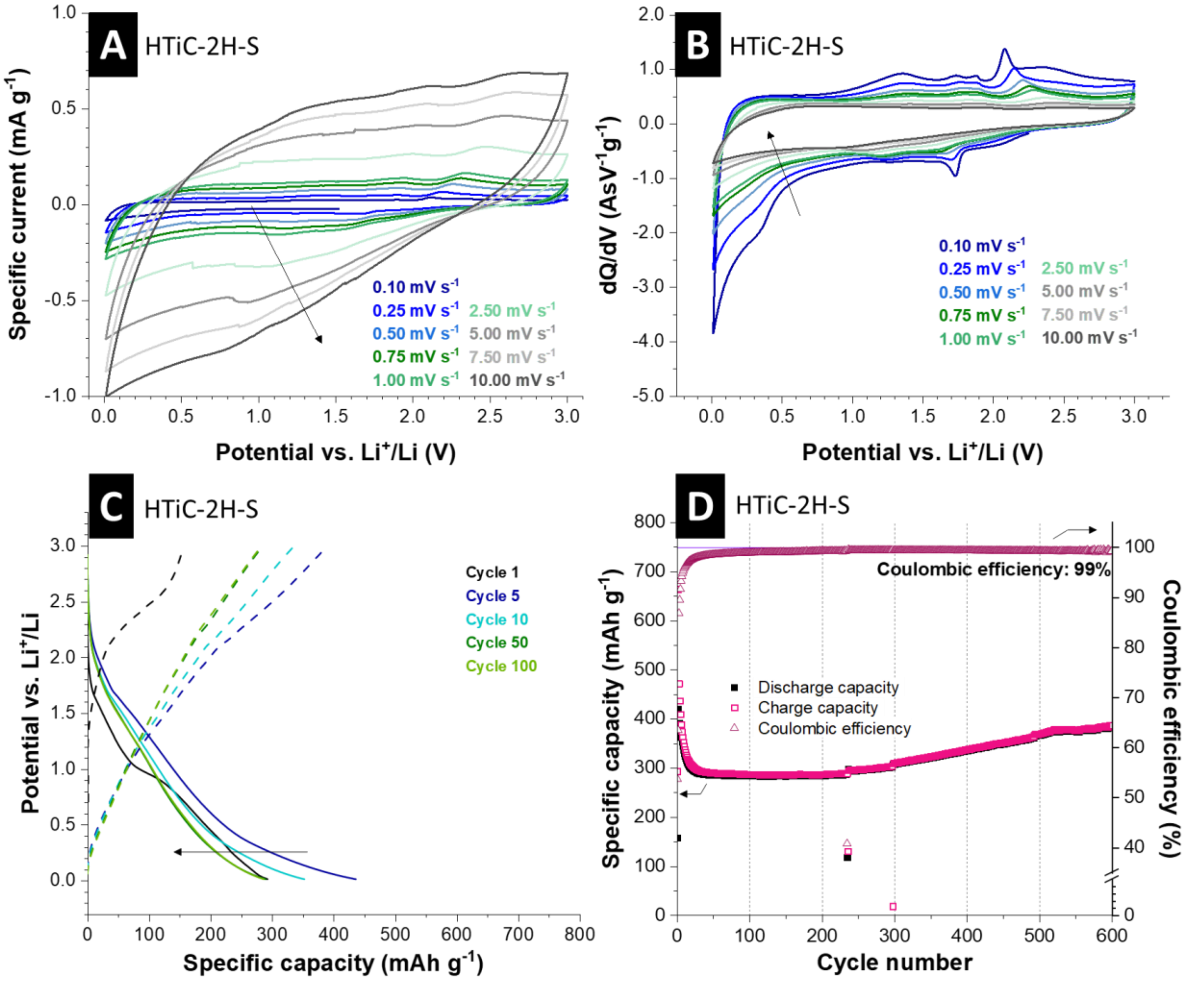

Figure S11: Electrochemical performance of sulfur-doped TiO_2-loaded carbon spherogels HTi-2H-S. **A** Cyclic voltammograms at different scanning rates, **B** Electrochemical performance showing cyclic voltammogram at different scanning rates and a potential range between 0.01 V and 3.00 V vs. Li^+/Li with normalization to the scan rate **C** Galvanostatic charge and discharge profiles between 0.01 V and 3.0 V vs. Li^+/Li, **D** Electrochemical cycling stability at a specific current of 0.25 A g^{-1} including corresponding Coulombic efficiency.

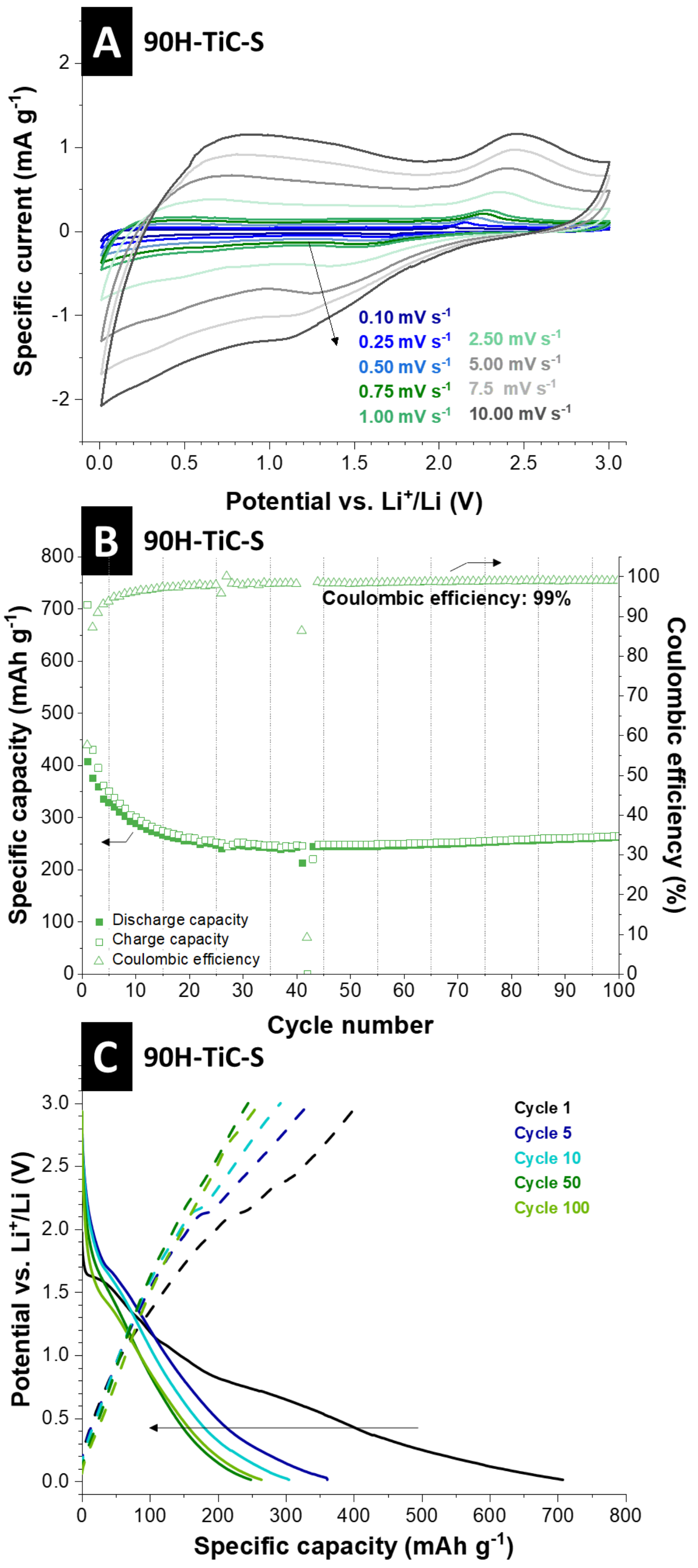

Figure S12: Electrochemical performance of sulfur-doped TiO_2-loaded carbon spherogels H-TiC-S. **A** Cyclic voltammograms at different scanning rates, **B** Electrochemical cycling stability at a specific current of 0.25 A g^{-1} including corresponding Coulombic efficiency, **C** Galvanostatic charge and discharge profiles between 0.01 V and 3.0 V vs. Li^+/Li.

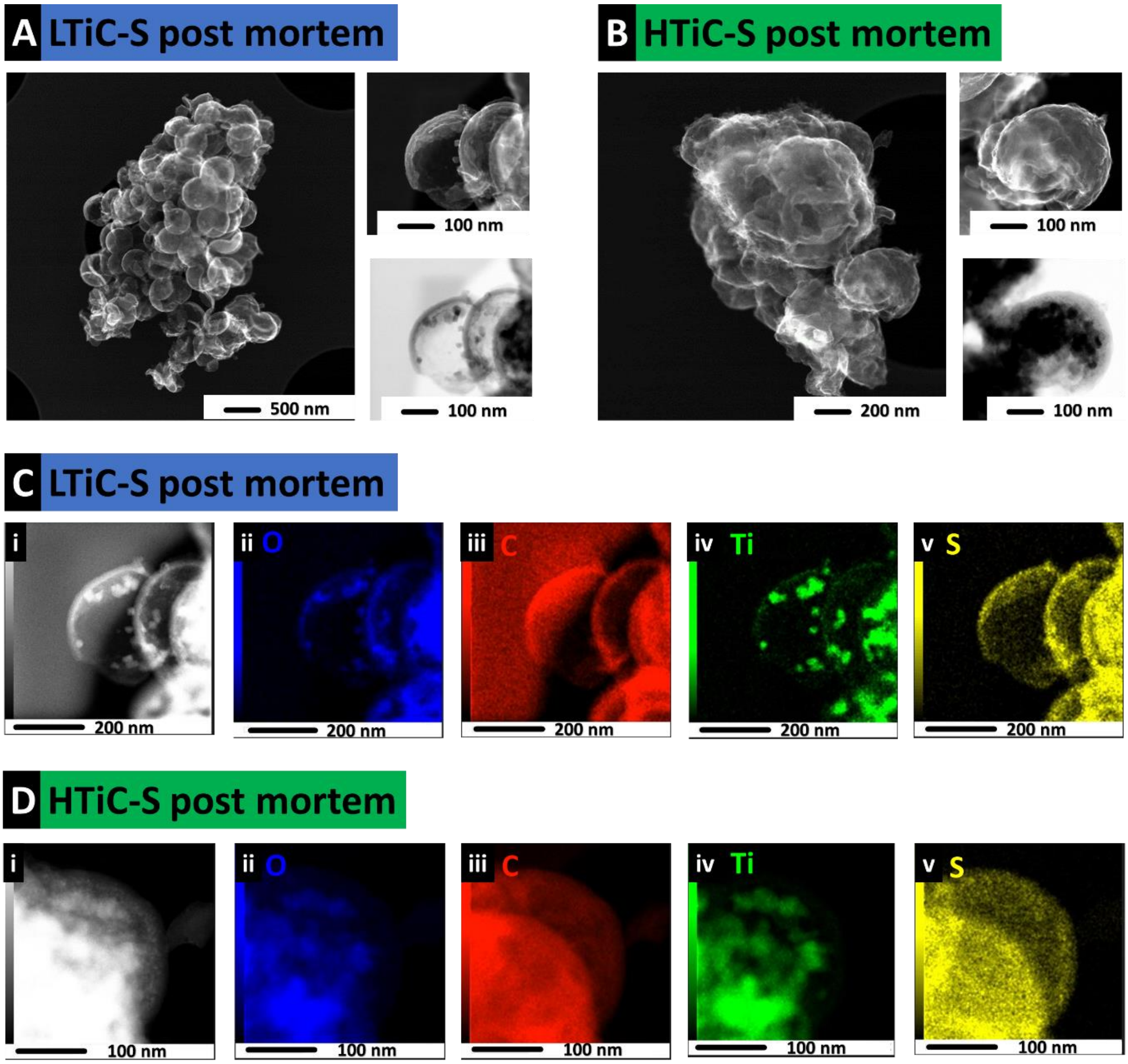

Figure S13: STEM images of post-mortem rate handling and their respective EDS analysis of LTiC-S in **A-B** and HTiC-S in **C-D**.
